# Correction: Subgingival Microbial Communities in Leukocyte Adhesion Deficiency and Their Relationship with Local Immunopathology

**DOI:** 10.1371/journal.ppat.1004860

**Published:** 2015-04-22

**Authors:** The PLOS Pathogens Staff

## Notice of Republication

This article was republished on April 6, 2015, to correct two errors with the Fig 5 legend. The errors were introduced during the typesetting process, and the publisher apologizes for the errors. Please download the article again and view the correct version. The originally published, uncorrected article and the republished, corrected article are provided here for reference.

## Supporting Information

S1 File Originally published, uncorrected article. (PDF)

S2 File Republished, corrected article. (PDF)
