# Supplementary material for: Subgingival Microbial Communities in Leukocyte Adhesion Deficiency and Their Relationship with Local Immunopathology
Source: PLoS Pathog. 2015 Mar 5;11(3):e1004698. doi: 10.1371/journal.ppat.1004698 (PMC4351202; doi:10.1371/journal.ppat.1004698)
Supplement: S1 Methods — (DOCX) [file ppat.1004698.s001.docx]

**S1 Methods**

**Comparison of prevalence for select species in LAD and Localized Aggressive Periodontitis**

Species highly prevalent for localized aggressive periodontitis (LAP) were retrieved from (Fine et al., Suppl Figure 3). Prevalence was defined as the average presence of a HOT over the entire LAD (n=5) or LAP (n=7) cohort multiplied by 100 to give percent prevalence.  A HOT was called present if it has a level of one or greater on the intensity scale.    In order to compare LAD prevalence with LAP prevalence, data were joined by probe ID on the HOMIM array.
