# Supplementary figures and images for: Subgingival Microbial Communities in Leukocyte Adhesion Deficiency and Their Relationship with Local Immunopathology
Source: PLoS Pathog. 2015 Mar 5;11(3):e1004698. doi: 10.1371/journal.ppat.1004698 (PMC4351202; doi:10.1371/journal.ppat.1004698)

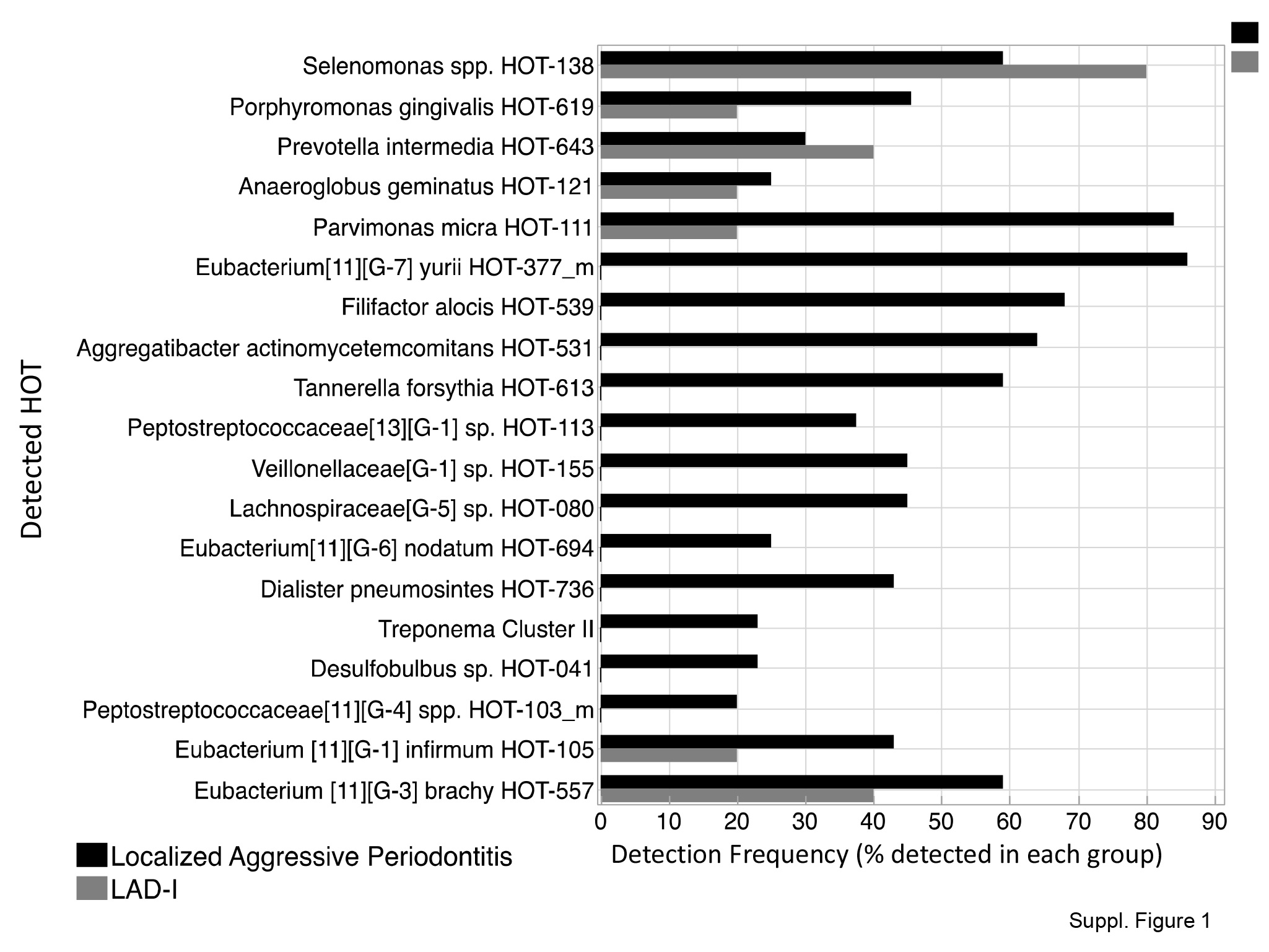

Supplement: S1 Fig — Frequency of detection of HOTs in the cohorts of localized aggressive periodontitis patients (LAP, n = 7, black) and LAD-I (n = 5, grey). HOTs shown are those highly detected in LAP patients only [16]. Frequency of Detection corresponds to % present in each cohort. (TIF) [file ppat.1004698.s002.tif]
