# Supplementary material for: Subgingival Microbial Communities in Leukocyte Adhesion Deficiency and Their Relationship with Local Immunopathology
Source: PLoS Pathog. 2015 Mar 5;11(3):e1004698. doi: 10.1371/journal.ppat.1004698 (PMC4351202; doi:10.1371/journal.ppat.1004698)
Supplement: S1 Table — (DOCX) [file ppat.1004698.s003.docx]

| **Patient ID** | **Age** | **Gender** | **Major Clinical Problems** | **Antibiotic Use** |  |
| --- | --- | --- | --- | --- | --- |
| mLAD1 | 14 | M | Otitis Media Infections  Skin ulcerative lesions  Pyoderma Gangrenosum  Major Oral Apthae | Prophylactic Antibiotic Use  (Trimethoprim-Sulfamethoxazole) | |
| mLAD2 | 12 | F | Mild clinical phenotype  Minimal secondary infections  Skin lesions  Pyoderma Gangrenosum | No antibiotics used for >6 months  Sporadic antibiotic use | |
| mLAD3 | 26 | M | Mild clinical phenotype  Minimal secondary infections  Folliculitis  Major Oral Apthae | No antibiotics used for >1 year  Sporadic antibiotic use | |
| mLAD4 | 38 | M | Skin lesions  Folliculitis  Major Oral Apthae | Prophylactic Antibiotics  (Amoxicillin/Clavulanate) | |
| sLAD | 13 | M | Recurrent Otitis Media  Gastroenteritis  Skin lesions  Cellulitis  Warts  Myositis | Prophylactic Antibiotics  (Amoxicillin/Clavulanate) | |

**TABLE SI**. **Clinical Information for the LAD cohort**
