# Supplementary material for: Subgingival Microbial Communities in Leukocyte Adhesion Deficiency and Their Relationship with Local Immunopathology
Source: PLoS Pathog. 2015 Mar 5;11(3):e1004698. doi: 10.1371/journal.ppat.1004698 (PMC4351202; doi:10.1371/journal.ppat.1004698)
Supplement: S2 Table — (DOCX) [file ppat.1004698.s004.docx]

TABLE S2 Probes with Multiple HOT Identities

| ProbeID | Full Name Probe ID |
| --- | --- |
| *Actinomyces meyeri* HOT-671_m | *Actinomyces meyeri* HOT-671 / *Actinomyces odontolyticus* HOT-701_AG08 |
| *Aggregatibacter segnis* HOT-762_m | *Aggregatibacter segnis* HOT-762 / *Aggregatibacter* sp. HOT-512_AA78 |
| *Campylobacter concisus* HOT-575_m | *Campylobacter concisus* HOT-575 / *Campylobacter rectus* HOT-748_T86 |
| *Campylobacter concisus* HOT-575_m | Campylobacter concisus HOT-575 / *Campylobacter rectus* HOT-748_X36 |
| *Campylobacter curvus* HOT-580_m | *Campylobacter curvus* HOT-580 / *Campylobacter rectus* HOT-748 / Campylobacter showae HOT-763_T87 |
| *Campylobacter curvus* HOT-580_m | *Campylobacter curvus* HOT-580 / *Campylobacter rectus* HOT-748 / *Campylobacter showae HOT*-763_X37 |
| *Capnocytophaga ochracea* HOT-700_m | *Capnocytophaga ochracea* HOT-700 / *Capnocytophaga* sp. HOT-323,326,864_Y83 |
| *Capnocytophaga sputigena* HOT-775_m | *Capnocytophaga sputigena* HOT-775 / *Capnocytophaga* sp. HOT-324_AG25 |
| *Catonella morbi* HOT-165_m | *Catonella morbi* HOT-165 / *Catonella* spp. HOT-164_O56 |
| *Eikenella corrodens* HOT-577_m | *Eikenella corrodens* HOT-577 / *Kingella denitrificans* HOT-582 / *Kingella* sp. HOT-012_AD98 |
| *Eubacterium* [11][G-7] *yurii* HOT-377_m | *Eubacterium* [11][G-7] *yurii* HOT-377 / *Peptostreptococcaceae* [11][G-7] sp. HOT-106_W84 |
| *Fusobacterium naviforme* HOT-689_m | *Fusobacterium naviforme* HOT-689 / *Fusobacterium nucleatum* ss *vincentii* HOT-200_M22 |
| *Gemella haemolysans* HOT-626_m | *Gemella haemolysans* HOT-626 / *Gemella sanguinis* HOT-757_K63 |
| Granulicatella adiacens HOT-534_m | *Granulicatella adiacens* HOT-534 / *Granulicatella elegans* HOT-596_W81 |
| *Klebsiella pneumoniae* HOT-731_m | *Klebsiella pneumoniae* HOT-731 / *Klebsiella pneumonia* ss *ozaenae* HOT-735_AG55 |
| *Lachnoanaerobaculum orale* HOT-082_m | *Lachnoanaerobaculum orale* HOT-082 / *Lachnoanaerobaculum saburreum* HOT-494_AB50 |
| *Lactobacillus casei* HOT-568_m | *Lactobacillus casei* HOT-568 / *Lactobacillus paracasei* HOT-716 / *Lactobacillus rhamnosus* HOT-749_W94 |
| *Lactobacillus gasseri* HOT-615_m | *Lactobacillus gasseri* HOT-615 / *Lactobacillus johnsonii* HOT-819_V86 |
| *Lactobacillus jensenii* HOT-839_m | *Lactobacillus jensenii* HOT-839 / *Lactobacillus salivarius* HOT-756_AB14 |
| *Leptotrichia hofstadii* HOT-224_m | *Leptotrichia hofstadii* HOT-224 / *Leptotrichia* sp. HOT-223_Y55 |
| *Leptotrichia wadei* HOT-222_m | *Leptotrichia wadei* HOT-222 / *Leptotrichia* sp. HOT-417_AA60 |
| *Neisseria gonorrhoeae* HOT-621_m | *Neisseria gonorrhoeae* HOT-621 / *Neisseria polysaccharea* HOT-737_O76 |
| *Neisseria oralis* HOT-014_m | *Neisseria oralis* HOT-014 / *Eikenella* sp. HOT-011_Y57 |
| *Porphyromonas catoniae* HOT-283_m | *Porphyromonas catoniae* HOT-283 / *Porphyromonas* sp. HOT-279_N95 |
| Porphyromonas endodontalis HOT-273_m | *Porphyromonas endodontalis* HOT-273 / *Porphyromonas* sp. HOT-285,395_W78 |
| *Prevotella histicola* HOT-298_m | *Prevotella histicola* HOT-298 / *Prevotella melaninogenica* HOT-469_T81 |
| *Prevotella loescheii* HOT-658_m | Prevotella loescheii HOT-658 / *Prevotella* sp. HOT-317,472_Y65 |
| *Prevotella pallens* HOT-714_m | *Prevotella pallens* HOT-714 / *Prevotella* sp. HOT-310_R67 |
| *Pseudomonas aeruginosa* HOT-536_m | *Pseudomonas aeruginosa* HOT-536 / *Pseudomonas otitidis* HOT-834 / *Pseudomonas* sp. HOT-032_AB68 |
| *Pseudomonas aeruginosa* HOT-536_m | *Pseudomonas aeruginosa* HOT-536 / *Pseudomonas otitidis* HOT-834_AB67 |
| *Rothia dentocariosa* HOT-587_m | *Rothia dentocariosa* HOT-587 */ Rothia mucilaginosa* HOT-681_E52 |
| *Selenomonas infelix* HOT-639_m | *Selenomonas infelix* HOT-639 / *Selenomonas* sp. HOT-126,479,481_AC13 |
| *Selenomonas infelix* HOT-639_m | *Selenomonas infelix* HOT-639 / *Selenomonas* sp. HOT-126,479,481_O54 |
| *Selenomonas noxia* HOT-130_m | *Selenomonas noxia* HOT-130 / *Selenomonas* sp. HOT-140_AC04 |
| *Streptococcus anginosus* HOT-543_m | *Streptococcus anginosus* HOT-543 / *Streptococcus gordonii* HOT-622_F49 |
| *Streptococcus anginosus* HOT-543_m | *Streptococcus anginosus* HOT-543 / *Streptococcus gordonii* HOT-622_X11 |
| *Streptococcus constellatus* HOT-576_m | *Streptococcus constellatus* HOT-576 / *Streptococcus intermedius* HOT-644_AB77 |
| *Streptococcus constellatus* HOT-576_m | Streptococcus constellatus HOT-576 / *Streptococcus intermedius* HOT-644_F48 |
| *Streptococcus infantis* HOT-638_m | *Streptococcus infantis* HOT-638 / *Streptococcus* sp. HOT-065_Y74 |
| *Streptococcus infantis* HOT-638_m | *Streptococcus infantis* HOT-638 / *Streptococcus* sp. HOT-065_AH35 |
| *Streptococcus mitis* bv2 HOT-398_m | *Streptococcus mitis* bv2 HOT-398 / *Streptococcus* sp. HOT-069_Q64 |
| *Streptococcus oralis* HOT-707_m | *Streptococcus oralis* HOT-707/ *Streptococcus* sp. HOT-064_F46 |
| *Streptococcus parasanguinis* I HOT-721_m | *Streptococcus parasanguinis* I HOT-721 / *Streptococcus parasanguinis* II HOT-411 / *Streptococcus* sp. HOT-057_R17 |
| *Streptococcus parasanguinis* I HOT-721_m | *Streptococcus parasanguinis* I HOT-721 / *Streptococcus parasanguinis* II HOT-411 / *Streptococcus* sp. HOT-057_V77 |
| *Streptococcus parasanguinis* I HOT-721_m | *Streptococcus parasanguinis* I HOT-721 / *Streptococcus* sp. HOT-057_AH37 |
| *Streptococcus salivarius* HOT-755_m | *Streptococcus salivarius* HOT-755 / *Streptococcus vestibularis* HOT-021 / *Streptococcus* sp. HOT-067_E34 |
| *Streptococcus salivarius* HOT-755_m | *Streptococcus salivarius* HOT-755 / *Streptococcus vestibularis* HOT-021_AH39 |
| TM7 [G-1] sp. HOT-347_m | TM7 [G-1] sp. HOT-347 / TM7 [G-2] sp. HOT-350_Y77 |
| *Veillonella dispar* HOT-160_m | *Veillonella dispar* HOT-160 / *Veillonella parvula* HOT-161_Q67 |
