# Supplementary material for: Subgingival Microbial Communities in Leukocyte Adhesion Deficiency and Their Relationship with Local Immunopathology
Source: PLoS Pathog. 2015 Mar 5;11(3):e1004698. doi: 10.1371/journal.ppat.1004698 (PMC4351202; doi:10.1371/journal.ppat.1004698)
Supplement: S3 Table — (DOCX) [file ppat.1004698.s005.docx]

TABLE S3 Cluster Identification for each HOT

| ProbeID | Cluster | Color | Health  (mean) | mLAD  (mean) | sLAD  (mean) |
| --- | --- | --- | --- | --- | --- |
| *Actinobaculum* sp. HOT-183 | 5 | Purple | 0.18 | 0.00 | 0.56 |
| *Leptotrichia buccalis* HOT-563 | 5 | Purple | 0.00 | 0.00 | 0.50 |
| *Prevotella oulora* HOT-288 | 5 | Purple | 0.35 | 0.18 | 0.50 |
| *Actinomyces gerencseriae* HOT-618 | 5 | Purple | 0.15 | 0.00 | 0.00 |
| *Eubacterium* [11][G-7] *yurii* HOT-377 | 5 | Purple | 0.15 | 0.00 | 0.00 |
| *Streptococcus constellatus* HOT-576 | 5 | Purple | 0.23 | 0.00 | 0.00 |
| *Bifidobacterium dentium* HOT-588 | 5 | Purple | 0.15 | 0.00 | 0.00 |
| *Leptotrichiaceae* [G-1] sp. HOT-210 | 5 | Purple | 0.15 | 0.00 | 0.00 |
| *Eubacterium* [11][G-6] *nodatum* HOT-694 | 5 | Purple | 0.23 | 0.00 | 0.00 |
| Fretibacterium Cluster | 5 | Purple | 0.15 | 0.00 | 0.00 |
| *Fretibacterium fastidiosum* HOT-363 | 5 | Purple | 0.15 | 0.00 | 0.00 |
| *Actinomyces* sp. HOT-170 | 5 | Purple | 0.44 | 0.00 | 0.00 |
| *Klebsiella pneumoniae* HOT-731_m | 5 | Purple | 0.31 | 0.00 | 0.00 |
| *Megasphaera micronuciformis* HOT-122 | 5 | Purple | 0.62 | 0.00 | 0.00 |
| *Ochrobactrum anthropi* HOT-544 | 5 | Purple | 0.38 | 0.00 | 0.00 |
| *Prevotella nigrescens* HOT-693 | 5 | Purple | 0.23 | 0.00 | 0.00 |
| *Tannerella forsythia* HOT-613 | 5 | Purple | 0.23 | 0.00 | 0.00 |
| *Treponema parvum* HOT-724 | 5 | Purple | 0.38 | 0.00 | 0.00 |
| *Actinomyces* sp. HOT-169 | 5 | Purple | 0.31 | 0.00 | 0.00 |
| *Capnocytophaga* sp. HOT-326 | 5 | Purple | 0.31 | 0.00 | 0.17 |
| *Dialister pneumosintes* HOT-736 | 5 | Purple | 0.23 | 0.00 | 0.00 |
| *Rothia mucilaginosa* HOT-681 | 5 | Purple | 0.38 | 0.14 | 0.00 |
| *Streptococcus salivarius* HOT-755_m | 5 | Purple | 0.54 | 0.00 | 0.00 |
| *Burkholderia* sp. HOT-406 | 5 | Purple | 0.15 | 0.18 | 0.33 |
| *Prevotella oris* HOT-311 | 5 | Purple | 0.31 | 0.18 | 0.00 |
| *Neisseria pharyngis* HOT-729 | 5 | Purple | 0.38 | 0.00 | 0.00 |
| *Veillonella* sp. HOT-780 | 5 | Purple | 0.31 | 0.18 | 0.00 |
| *Bacteroidales* [G-2] sp. HOT-274 | 5 | Purple | 0.05 | 0.00 | 1.33 |
| *Selenomonas* sp. HOT-136 | 5 | Purple | 0.00 | 0.09 | 1.00 |
| *Pseudomonas aeruginosa* HOT-536_m | 5 | Purple | 0.00 | 0.32 | 1.00 |
| *Pseudomonas otitidis*&*aeruginosa&*HOT-834 | 5 | Purple | 0.00 | 0.27 | 1.00 |
| Pseudomonas Cluster | 5 | Purple | 0.23 | 0.64 | 1.67 |
| *Prevotella loescheii* HOT-658_m | 5 | Purple | 0.23 | 0.73 | 1.67 |
| *Actinomyces* sp. HOT-175 | 5 | Purple | 0.23 | 0.45 | 0.00 |
| *Capnocytophaga sputigena* HOT-775_m | 5 | Purple | 0.31 | 0.36 | 0.00 |
| *Leptotrichia wadei* HOT-222_m | 5 | Purple | 0.00 | 0.45 | 0.00 |
| SR1 [G-1] sp. HOT-345 | 5 | Purple | 0.00 | 0.23 | 0.00 |
| *Selenomonas flueggei* HOT-125 | 5 | Purple | 0.08 | 0.27 | 0.00 |
| TM7 [G-1] sp. HOT-346 | 5 | Purple | 0.04 | 0.59 | 0.17 |
| TM7 [G-1] sp. HOT-347_m | 5 | Purple | 0.00 | 0.82 | 0.67 |
| *Peptostreptococcus stomatis* HOT-112 | 5 | Purple | 0.31 | 0.64 | 0.00 |
| *Selenomonas infelix* HOT-639_m | 5 | Purple | 0.27 | 0.59 | 0.00 |
| *Veillonella atypica* HOT-524 | 5 | Purple | 0.77 | 0.18 | 0.00 |
| Actinomyces Cluster | 5 | Purple | 0.00 | 0.45 | 0.33 |
| *Streptococcus parasanguinis* I HOT-721_m | 5 | Purple | 0.23 | 0.36 | 0.00 |
| *Oribacterium* sp. HOT-078 | 5 | Purple | 0.00 | 0.23 | 0.67 |
| *Prevotella melaninogenica* HOT-469 | 5 | Purple | 0.08 | 0.18 | 0.00 |
| *Prevotella histicola* HOT-298_m | 5 | Purple | 0.31 | 0.36 | 0.00 |
| *Lactobacillus casei* HOT-568_m | 5 | Purple | 0.00 | 0.55 | 0.00 |
| *Solobacterium moorei* HOT-678 | 5 | Purple | 0.15 | 0.59 | 0.00 |
| *Scardovia wiggsiae* HOT-195 | 5 | Purple | 0.00 | 0.73 | 0.00 |
| *Neisseria gonorrhoeae* HOT-621_m | 5 | Purple | 0.62 | 1.09 | 0.00 |
| *Aggregatibacter segnis* HOT-762_m | 5 | Purple | 0.38 | 0.64 | 0.00 |
| *Stomatobaculum* sp. HOT-097 | 5 | Purple | 0.00 | 0.82 | 0.00 |
| *Capnocytophaga ochracea* HOT-700_m | 5 | Purple | 0.38 | 1.00 | 0.00 |
| *Prevotella* sp. HOT-299 | 5 | Purple | 0.46 | 1.64 | 0.00 |
| *Neisseria flavescens* HOT-610 | 5 | Purple | 0.54 | 0.91 | 0.00 |
| *Streptococcus infantis* HOT-638_m | 5 | Purple | 0.27 | 1.23 | 0.00 |
| *Actinomyces meyeri* HOT-671_m | 5 | Purple | 0.00 | 0.00 | 1.33 |
| *Treponema* sp. HOT-231 | 5 | Purple | 0.08 | 0.00 | 1.33 |
| *Lachnospiraceae* [G-3] sp. HOT-100 | 5 | Purple | 0.38 | 0.27 | 1.33 |
| *Capnocytophaga* sp. HOT-335 | 5 | Purple | 0.38 | 0.00 | 2.00 |
| *Treponema maltophilum* HOT-664 | 5 | Purple | 0.23 | 0.00 | 2.00 |
| *Treponema* sp. HOT-257 | 5 | Purple | 0.15 | 0.18 | 1.33 |
| *Porphyromonas gingivalis* HOT-619 | 5 | Purple | 0.46 | 0.18 | 1.33 |
| *Prevotella intermedia* HOT-643 | 5 | Purple | 0.26 | 0.06 | 1.00 |
| Prevotella Cluster II | 5 | Purple | 0.38 | 0.45 | 1.00 |
| *Treponema denticola* HOT-584 | 5 | Purple | 0.77 | 0.00 | 1.33 |
| *Eubacterium* [11][G-3] *brachy* HOT-557 | 5 | Purple | 0.31 | 0.18 | 3.17 |
| *Porphyromonas endodontalis* HOT-273_m | 5 | Purple | 0.23 | 0.00 | 2.67 |
| *Parvimonas micra* HOT-111 | 5 | Purple | 0.77 | 0.00 | 2.83 |
| *Abiotrophia defectiva* HOT-389 | 4 | Green | 1.00 | 0.36 | 0.33 |
| *Porphyromonas* sp. HOT-279 | 4 | Green | 1.31 | 0.00 | 0.00 |
| *Brevundimonas diminuta* HOT-590 | 4 | Green | 0.92 | 0.00 | 0.00 |
| *Gemella morbillorum* HOT-046 | 4 | Green | 0.92 | 0.00 | 0.00 |
| *Granulicatella elegans* HOT-596 | 4 | Green | 1.35 | 0.09 | 0.00 |
| *Haemophilus* sp. HOT-036 | 4 | Green | 1.38 | 0.00 | 0.00 |
| *Capnocytophaga gingivalis* HOT-337 | 4 | Green | 0.92 | 0.18 | 0.83 |
| *Corynebacterium durum* HOT-595 | 4 | Green | 0.54 | 0.36 | 0.00 |
| *Rothia dentocariosa* HOT-587 | 4 | Green | 1.33 | 0.00 | 0.00 |
| *Streptococcus* sp. HOT-070 | 4 | Green | 0.92 | 0.00 | 0.00 |
| *Eikenella corrodens* HOT-577_m | 4 | Green | 1.69 | 0.91 | 1.33 |
| *Kingella denitrificans* HOT-582 | 4 | Green | 1.31 | 0.14 | 0.50 |
| *Lautropia mirabilis* HOT-022 | 4 | Green | 1.15 | 0.27 | 1.00 |
| *Streptococcus constellatus* HOT-576_m | 4 | Green | 1.69 | 0.32 | 3.17 |
| *Capnocytophaga granulosa* HOT-325 | 4 | Green | 0.92 | 0.09 | 0.00 |
| *Capnocytophaga leadbetteri* HOT-329 | 4 | Green | 0.67 | 0.09 | 0.11 |
| *Cardiobacterium hominis* HOT-633 | 4 | Green | 0.81 | 0.00 | 0.00 |
| *Lachnospiraceae* [G-2] sp. HOT-096 | 4 | Green | 0.62 | 0.00 | 0.00 |
| *Porphyromonas catoniae* HOT-283_m | 4 | Green | 0.69 | 0.00 | 0.00 |
| *Eubacterium* [11][G-7] *yurii* HOT-377_m | 4 | Green | 0.92 | 0.00 | 0.00 |
| *Streptococcus mitis* bv2 HOT-398 | 4 | Green | 0.62 | 0.00 | 1.00 |
| *Streptococcus mitis* bv2 HOT-398_m | 4 | Green | 0.92 | 0.00 | 0.67 |
| *Actinomyces naeslundii* HOT-176 | 3 | Blue | 1.69 | 1.64 | 0.33 |
| *Campylobacter concisus* HOT-575 | 3 | Blue | 0.81 | 1.09 | 1.17 |
| *Selenomonas noxia* HOT-130_m | 3 | Blue | 0.31 | 1.45 | 1.33 |
| *Lachnoanaerobaculum orale* HOT-082_m | 3 | Blue | 0.62 | 2.00 | 1.33 |
| *Campylobacter gracilis* HOT-623 | 3 | Blue | 0.85 | 1.36 | 3.00 |
| *Campylobacter curvus* HOT-580_m | 3 | Blue | 0.62 | 1.05 | 1.50 |
| *Corynebacterium matruchotii* HOT-666 | 3 | Blue | 1.31 | 0.59 | 0.83 |
| *Propionibacterium propionicum* HOT-739 | 3 | Blue | 0.50 | 0.86 | 1.00 |
| *Streptococcus cristatus* HOT-578 | 3 | Blue | 0.77 | 0.73 | 1.00 |
| *Lachnoanaerobaculum saburreum* HOT-494 | 3 | Blue | 0.38 | 0.73 | 1.00 |
| *Selenomonas* sp. HOT-137 | 3 | Blue | 0.15 | 0.91 | 1.33 |
| *Selenomonas* sp. HOT-138 | 3 | Blue | 0.23 | 0.82 | 2.33 |
| *Bergeyella* sp. HOT-322 | 2 | Brown | 2.08 | 0.91 | 0.67 |
| *Granulicatella adiacens* HOT-534 | 2 | Brown | 1.38 | 1.64 | 0.00 |
| *Granulicatella adiacens* HOT-534_m | 2 | Brown | 1.62 | 1.73 | 0.00 |
| *Streptococcus anginosus* HOT-543_m | 2 | Brown | 1.15 | 0.68 | 0.00 |
| *Streptococcus australis* HOT-073 | 2 | Brown | 2.15 | 0.55 | 0.00 |
| *Gemella haemolysans* HOT-626 | 2 | Brown | 3.08 | 1.18 | 3.00 |
| *Gemella haemolysans* HOT-626_m | 2 | Brown | 2.46 | 0.64 | 0.00 |
| *Veillonella dispar* HOT-160_m | 2 | Brown | 2.54 | 1.45 | 1.00 |
| *Haemophilus parainfluenzae* HOT-718 | 2 | Brown | 3.77 | 3.18 | 1.67 |
| *Streptococcus oralis* HOT-707_m | 2 | Brown | 4.23 | 1.55 | 1.00 |
| *Rothia dentocariosa* HOT-587_m | 2 | Brown | 3.62 | 0.82 | 0.67 |
| *Campylobacter concisus* HOT-575_m | 1 | Black | 1.92 | 2.86 | 3.17 |
| *Kingella oralis* HOT-706 | 1 | Black | 2.23 | 2.68 | 1.33 |
| *Campylobacter showae* HOT-763 | 1 | Black | 1.46 | 2.05 | 3.00 |
| *Capnocytophaga sputigena* HOT-775 | 1 | Black | 1.08 | 1.91 | 2.33 |
| *Catonella morbi* HOT-165_m | 1 | Black | 1.08 | 0.91 | 3.00 |
| Fusobacterium Cluster | 1 | Black | 2.77 | 1.55 | 3.67 |
| *Fusobacterium periodonticum* HOT-201 | 1 | Black | 2.15 | 1.18 | 0.33 |
| Neisseria Cluster | 1 | Black | 1.38 | 1.91 | 1.67 |
| *Neisseria elongata* HOT-598 | 1 | Black | 2.00 | 2.27 | 2.00 |
