# Supplementary material for: Correction: Subgingival Microbial Communities in Leukocyte Adhesion Deficiency and Their Relationship with Local Immunopathology
Source: PLoS Pathog. 2015 Apr 22;11(4):e1004860. doi: 10.1371/journal.ppat.1004860 (PMC4406857; doi:10.1371/journal.ppat.1004860)
Supplement: S1 File — (PDF) [file ppat.1004860.s001.pdf]

PLOS | PATHOGENS

RESEARCH ARTICLE

Subgingival Microbial Communities in Leukocyte Adhesion Deficiency and Their Relationship with Local Immunopathology

Niki M. Moutsopoulos[1]*, Natalia I. Chalmers[2☯], Jennifer J. Barb[3☯], Loreto Abusleme[1], Teresa Greenwell-Wild[1], Nicolas Dutzan[1], Bruce J. Paster[4,5], Peter J. Munson[3], Daniel H. Fine[6], Gulbu Uzel[7], Steven M. Holland[7]

1 Oral Immunity and Inflammation Unit, Oral and Pharyngeal Cancer Branch, National Institute of Dental and Craniofacial Research, National Institutes of Health, Bethesda, Maryland, United States of America, **2** Clinical Research Core, National Institute of Dental and Craniofacial Research, National Institutes of Health, Bethesda, Maryland, United States of America, **3** Center for Information Technology, National Institutes of Health, Bethesda, Maryland, United States of America, **4** The Forsyth Institute, Cambridge, Massachusetts, United States of America, **5** Harvard School of Dental Medicine, Boston, Massachusetts, United States of America, **6** Rutgers School of Dental Medicine, Rutgers University, Newark, New Jersey, United States of America, **7** National Institute of Allergy and Infectious Diseases, Laboratory of Clinical Infectious Diseases, National Institutes of Health, Bethesda, Maryland, United States of America

☯ These authors contributed equally to this work.
* nmoutsop@mail.nih.gov

OPEN ACCESS

Citation: Moutsopoulos NM, Chalmers NI, Barb JJ, Abusleme L, Greenwell-Wild T, Dutzan N, et al. (2015) Subgingival Microbial Communities in Leukocyte Adhesion Deficiency and Their Relationship with Local Immunopathology. PLoS Pathog 11(3): e1004698. doi:10.1371/journal.ppat.1004698

Editor: Andrew L Goodman, Yale University, UNITED STATES

Received: September 17, 2014

Accepted: January 22, 2015

Published: March 5, 2015

Copyright: This is an open access article, free of all copyright, and may be freely reproduced, distributed, transmitted, modified, built upon, or otherwise used by anyone for any lawful purpose. The work is made available under the Creative Commons CC0 public domain dedication.

Data Availability Statement: All relevant data are within the paper and its Supporting Information files.

Funding: This research was supported in part by the Intramural Research Program of the NIH, National Institute of Dental and Craniofacial Research (NIDCR) and National Institute of Allergy and Infectious Diseases (NIAID). The funders had no role in study design, data collection and analysis, decision to publish, or preparation of the manuscript.

Abstract

Leukocyte Adhesion Deficiency I (LAD-I) is a primary immunodeficiency caused by single gene mutations in the CD18 subunit of β2 integrins which result in defective transmigration of neutrophils into the tissues. Affected patients suffer from recurrent life threatening infections and severe oral disease (periodontitis). Microbial communities in the local environment (subgingival plaque) are thought to be the triggers for inflammatory periodontitis, yet little is known regarding the microbial communities associated with LAD-I periodontitis. Here we present the first comprehensive characterization of the subgingival communities in LAD-I, using a 16S rRNA gene-based microarray, and investigate the relationship of this tooth adherent microbiome to the local immunopathology of periodontitis. We show that the LAD subgingival microbiome is distinct from that of health and Localized Aggressive Periodontitis. Select periodontitis-associated species in the LAD microbiome included *Parvimonas micra*, *Porphyromonas endodontalis*, *Eubacterium brachy* and *Treponema species*. *Pseudomonas aeruginosa*, a bacterium not typically found in subgingival plaque is detected in LAD-I. We suggest that microbial products from LAD-associated communities may have a role in stimulating the local inflammatory response. We demonstrate that bacterial LPS translocates into the lesions of LAD-periodontitis potentially triggering immunopathology. We also show in *in vitro* assays with human macrophages and *in vivo* in animal models that microbial products from LAD-associated subgingival plaque trigger IL-23-related immune responses, which have been shown to dominate in patient lesions. In conclusion, our current study characterizes the subgingival microbial communities in LAD-periodontitis and supports their role as triggers of disease pathogenesis.

Competing Interests: The authors have declared that no competing interests exist.

Author Summary

Leukocyte adhesion deficiency (LAD) is a primary immunodeficiency resulting from gene mutations in the CD18 subunit of β2 integrins that lead to defective neutrophil adhesion and transmigration into tissues. Affected patients suffer from recurrent life threatening infections and from a severe form of the oral disease periodontitis. The setting of this rare monogenic immune disorder provides a unique opportunity to explore consequences of defective neutrophil tissue transmigration on immunity and microbial colonization in barrier sites such as the oral mucosa. Furthermore, characterization of the oral- subgingival microbiome in LAD expands our understanding of LAD periodontitis, an aggressive disease which is recalcitrant to treatment and often leads to loss of the entire dentition in adolescence. Our current studies in a cohort of LAD patients show that the subgingival microbiome in LAD- periodontitis is unique in its composition and differs from that of health and aggressive periodontitis. Notably our studies reveal that the subgingival communities of LAD can serve as initial triggers for local immunopathology through translocation of bacterial products into tissues and stimulation of local IL-23-related destructive inflammatory responses.

Introduction

Leukocyte Adhesion Deficiency type I (LAD-I) is a rare autosomal recessive disorder with an estimated prevalence of one in 100,000 births. It is caused by mutations in the CD18 (*ITGB2*) gene, which is the common chain of β2 integrin molecules, involved in the formation of all β2 integrins (CD11a/CD18, CD11b/CD18, CD11c/CD18 and CD11d/CD18) [1,2]. Defects in CD18 expression lead to either very low or no expression of associated β2 integrins. As a result patients have defective neutrophil adhesion and transmigration into tissues. Affected individuals suffer from recurrent infections and invariably develop early-onset generalized aggressive periodontitis featuring severe bone loss and premature loss of primary and permanent teeth [1,3].

LAD-I is suspected based on delayed umbilical stump separation and recurrent infections. The diagnosis is made by measuring the level of expression of CD18 on peripheral neutrophils and confirmed by identifying a CD18 mutation [4]. The clinical presentation of LAD-I can be severe (sLAD) or moderate (mLAD) and relates to the remaining level of CD18 expression and/or function on neutrophils. Features of severe deficiency (very low CD18 expression/function) include delayed umbilical stump separation, umbilical stump infection, persistent leukocytosis in the absence of active infection (>15,000/μL), and severe recurrent infections. Infections of the skin, upper and lower airways, bowel, and perirectal area and septicemia usually due to *Staphylococcus aureus* or gram-negative rods, most notably *Pseudomonas* species, are common [5]. Severe destructive periodontitis is also a hallmark of LAD disease [5]. Patients with severe LAD-I often develop complete bone loss around teeth early in life, and may lose their entire dentition during adolescence, only a few years after the transition to permanent dentition [6]. Patients with moderate deficiency are usually diagnosed later in life, have normal umbilical stump separation, and have fewer life-threatening infections. These patients also have leukocytosis, delayed wound healing and moderate periodontal disease when compared to the severe LAD-I patients [3]. Strikingly, the severity of periodontitis in LAD patients has been shown to inversely correlate with CD18 expression on peripheral neutrophils [6].

Until recently LAD-I periodontitis was considered an aggressive infection, resulting from defective neutrophil surveillance of local tissues [7]. However, our recent investigations of the

molecular mechanisms showed absence of tissue invasive infection. Instead, we documented dysregulated IL-23/IL-17 inflammatory responses driving periodontal destruction in LAD periodontitis [6]. Triggers for the initiation of the inflammatory response are considered the local bacteria and their byproducts, represented in periodontitis by the tooth adherent microbial biofilm, yet this has not conclusively been shown. In LAD-I these microbial-stimulated responses become unrestrained due to the lack of regulatory mechanisms that require the presence of neutrophils in the tissues [6].

To date, the composition of the bacterial communities associated with LAD-I periodontitis has not been comprehensively characterized. The context of LAD-I provides a unique opportunity to evaluate the consequences of CD18 deficiency and lack of neutrophil transmigration in a human disease, specifically periodontitis. How bacterial colonization in the oral cavity and particularly at the site of periodontitis may be altered in the absence of neutrophil surveillance is an important question.

To this end, we sought to characterize the composition of the subgingival microbiome in a cohort of LAD-I patients and evaluate the potential of LAD-I microbial communities to stimulate pro-inflammatory IL-17-related responses.

Results

Patients with LAD-I exhibit severe periodontitis

One of the hallmarks of LAD-I is severe periodontitis at a young age, which often leads to loss of the entire dentition despite treatment [2,6]. We evaluated five patients with LAD-I, (diagnosed by medical history, CD18 mutation and low CD18 expression of peripheral neutrophils) for the presence of periodontal disease. All five patients were diagnosed with generalized moderate to severe periodontitis, based on measurements of bone loss (Probing depth = PD and Clinical attachment loss = CAL) [8]. Severity of periodontitis correlated with the level of neutrophil dysfunction [6] and severity of general clinical manifestations (S1 Table). Of our patients, one had exceptionally severe periodontitis (sLAD) and required full mouth extractions shortly after initial protocol enrollment at the age of 13. All other patients were diagnosed with moderate-severe periodontitis based on official classification criteria but had mild/moderate levels of disease (mLAD) compared to our severe case (sLAD). Patients with mLAD had some variation in levels of bone and tooth loss, probably reflecting progression of disease with age. LAD patients were exposed to antibiotics either sporadically or continuously depending on the severity of their clinical picture (S1 Table). Our healthy control subjects were systemically healthy young adults without detectable periodontitis, gingivitis or any significant oral findings (clinical characteristics described in Table 1).

LAD-I subgingival microbiome is distinct

Subgingival microbial communities in LAD-I are closely associated with an aggressive disease process, therefore we were interested in characterizing the nature of the subgingival microbiome in LAD. We first investigated the total microbial biomass in LAD-I and health. For this purpose, we quantified bacterial load in subgingival samples using a 16S rRNA gene based real time PCR assay and found that LAD-I patients had significantly higher bacterial burden than did healthy individuals, as earlier seen in patients and mice mimicking the LAD phenotype [6] (Fig. 1A).

Next, we characterized the microbial composition of LAD-I associated communities. We performed analysis of the subgingival microbiome using a comprehensive 16S rRNA gene-based microarray (Human Oral Microbe Identification Microarray, HOMIM) which allows for the simultaneous detection of about 300 of the most prevalent oral bacterial species, including

Table 1. Patient cohort characterization.

Patient ID	Age	Gender	Full Mouth CAL*	PD# mm (site)	Missing Teeth	CD18 Mutation
H1	21	F	1.9 ± 0.08	< 3 mm	0	N/A
H2	23	F	2.2 ± 0.17	< 3 mm	0	N/A
H3	23	F	1.8 ± 0.02	< 3 mm	0	N/A
H4	24	M	2 ± 0.09	< 3 mm	0	N/A
H5	29	F	1.6 ± 0.02	< 3 mm	0	N/A
H6	23	M	2.4 ± 0.12	< 3 mm	0	N/A
H7	22	F	2.7 ± 0.15	< 3mm	0	N/A
H8	22	F	2.3 ± 0.21	< 3mm	0	N/A
H9	29	F	1.6 ± 0.02	< 3 mm	0	N/A
H10	23	M	2.4 ± 0.12	< 3 mm	0	N/A
H11	26	M	2.8 ± 0.22	< 3 mm	0	N/A
H12	28	M	1.7 ± 0.17	< 3 mm	0	N/A
mLAD1	14	M	3.6 ± 0.31	3–4mm	0	850G(R)A; G284S and C2070 del T; fs transmembrane domain
mLAD2	12	F	3 ± 0.12	3–4mm	0	Null allele (specific mutation unknown);C2070 del T; fs transmembrane domain
mLAD3	26	M	4.5 ± 0.23	5–6mm	8	
mLAD4	38	M	4.8 ± 0.34	5–6mm	20	1052 A→G; N351S and 741–14C→ A; 247+ PSSQ
sLAD	13	M	7.2 ± 0.72	>7mm	4	Homozygous del exon 12,13

*CAL = Clinical Attachment Loss (clinical measurement of periodontitis)

PD = Probing Depth (clinical measurement of periodontitis)

doi:10.1371/journal.ppat.1004698.t001

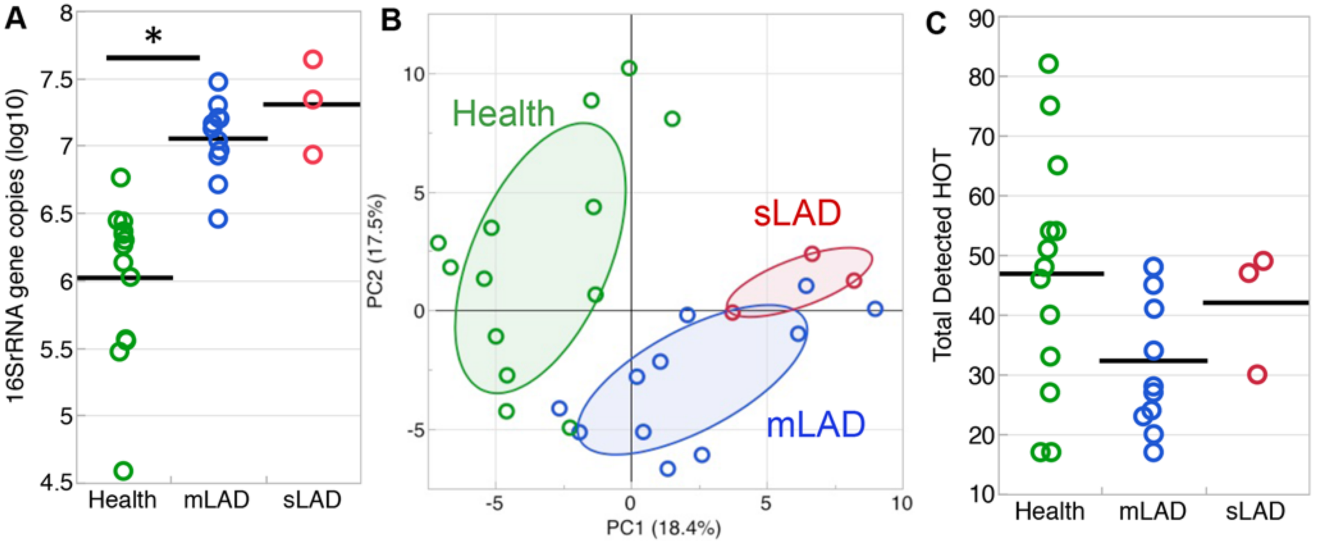

Fig 1. Microbial load and diversity of LAD-1 subgingival communities. (A) Total bacterial load (quantified with real-time PCR for 16S rRNA) shown in health (green), mild/moderate LAD (mLAD, blue) and severe LAD (sLAD, red). Bacterial load values are expressed as log (10) of 16S rRNA gene copy number and each circle represents a site. Mean values are indicated with a line. * indicates $p<0.05$. (B) Principal component analysis based on detection levels for bacterial taxa in the HOMIM microarray. Principal component 1 (PC1, x-axis, 18.4%) vs. principal component 2 (PC2, y-axis, 17.5%) account for 35.9% of the total data variability. Microbial communities from health (green circles) cluster away from LAD-I communities (mLAD, blue circles and sLAD, red circles). Ellipse is drawn around 50% of the data within the group. (C) Total number of HOT detected per group in health (green), mild/moderate LAD (blue) and severe LAD (red). Each circle represents a site. Mean values are indicated with a line. Multiple sites from the same patient were first averaged to obtain one value per patient prior to calculating mean values both for (A) and (C).

doi:10.1371/journal.ppat.1004698.g001

many known periodontal pathogens and other not-yet-cultivated species, but also select non-oral species. Principal component analysis (PCA) of level of detection for the total microarray data was performed. Visual inspection of Principal Component 1 (PC1) vs Principal Component 2 (PC2) shows that the LAD-I samples cluster away from those of health. Samples from our sLAD-I patient along with a few of the mLAD samples were furthest away from health (Fig. 1B). This first visual representation of the microbial data might suggest that LAD-periodontitis harbors a unique microbiome which is distinct from health, particularly in its severe forms.

To assess if this clear distinction can be attributed to a differential number of detected species between groups, we examined the total number of species present within the microbial communities. Although the sLAD and mLAD samples had higher total bacterial loads (Fig. 1A), there were no significant differences in the total numbers of Human Oral Taxon/Taxa (HOT used interchangeably with the term species throughout the manuscript) detected between the groups (Fig. 1C). In fact, LAD communities had fewer species detected on average, unlike the microbial communities typically detected in chronic and aggressive periodontitis which have been reported to present with an increased complexity [9]. Notable also was the great variability between health-associated communities, reflecting variability of microbial colonization in health.

Characterization of the LAD-I subgingival microbiome

To further analyze the microbial composition in LAD-I periodontitis and health, we performed unsupervised two-way hierarchical clustering of the detection level data for each of the species on the microarray. This unbiased approach yielded a clear separation between the health and LAD-I microbiomes, visible on the x-axis on the hierarchical diagram (Fig. 2). The y-axis on the hierarchical diagram shows the species detected, which grouped in five distinct clusters. Clusters 1 and 2 included species shared at varying levels of detection between health and LAD-I and could be considered to be part of a "shared microbiome". Species detected at higher levels or prevalence in the LAD-I group and particularly in sLAD were found within Cluster 3 and Cluster 5. Finally, the most striking observation was the absence of multiple HOT in LAD-I, particularly noticeable in Cluster 4 bacterial taxa. A complete list of all HOT and their mean detection levels per group is presented in S3 Table.

We first wanted to document the bacterial species present in health and LAD-I, without taking into consideration their level of detection. For this purpose, we created a Venn diagram displaying the unique species detected per group, as well as the species shared between groups (Fig. 3). Species shared between groups represented a "shared microbiome". Twenty-seven species were shared between health, severe and moderate LAD, 31 species shared between health and mLAD and 7 species shared between health and sLAD. Highly shared species (represented also in Clusters 1 and 2 of the hierarchical diagram) included *Kingella oralis* HOT-706, *Campylobacter concius* HOT-575_m, *Streptococcus oralis* HOT-707_m, *Neisseria elongata* HOT-598 and *Haemophilus parainfluenzae* HOT-718. Species present in only one group included 6 unique HOT for mLAD, 10 for sLAD, 4 in both moderate and severe LAD and 40 HOT detected only in health, reflecting depletion of a high number of health-associated species in the LAD microbiome. Species present in health and absent in LAD included a number of health associated bacteria of the *Streptococcus* and *Actinomyces* genus, but also species such as *Porphyromonas catoniae* HOT-283 and *Rothia dentocariosa*, previously associated with health [9]. Interestingly, a few species typically enriched in periodontitis were present (albeit at low levels) in health such as *Eubacterium* [11][G-6] *nodatum* and *Tannerella forsythia*.

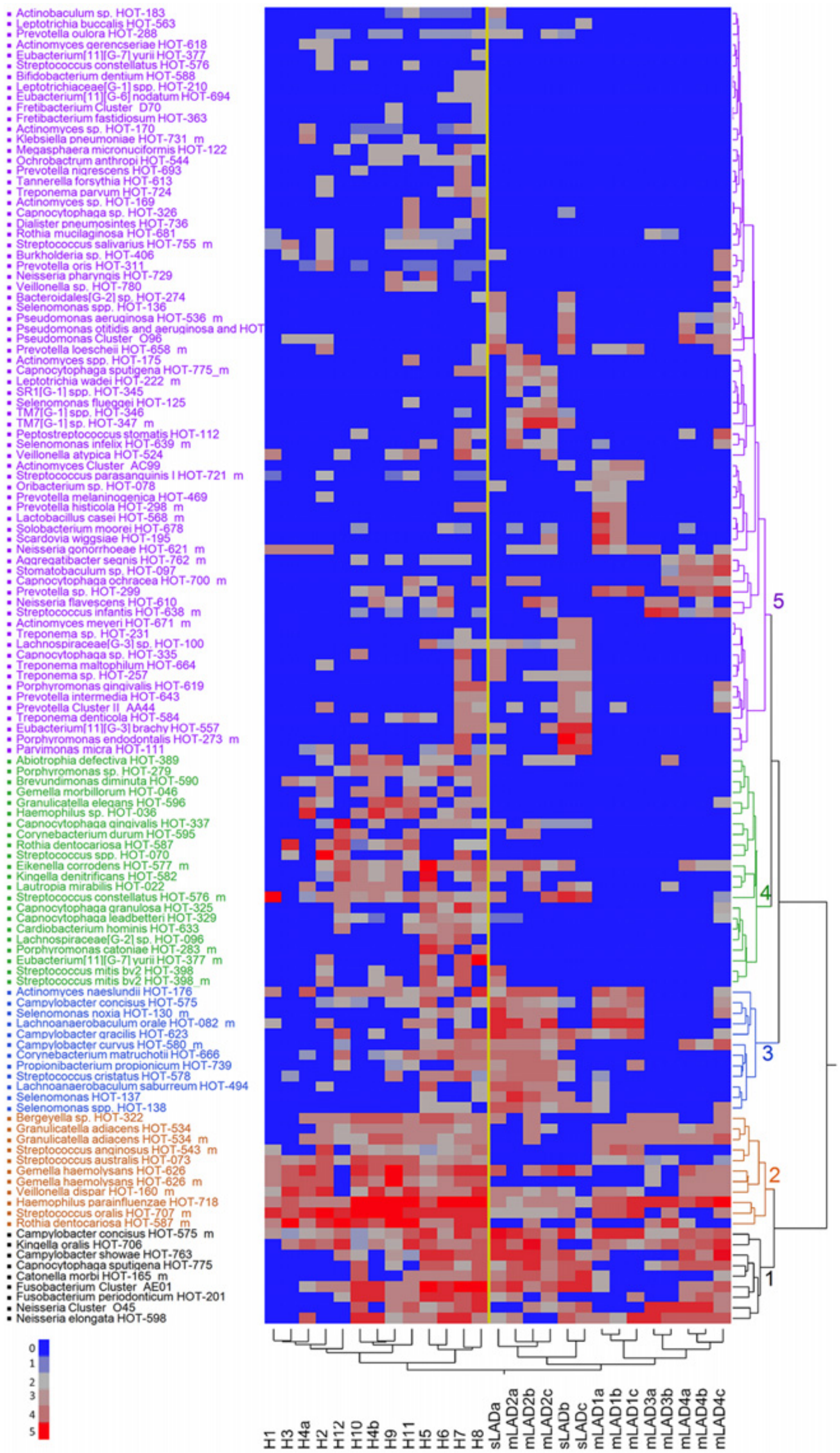

Fig 2. Detection levels of subgingival microbes in health and LAD. Two way hierarchical cluster analysis with Ward's distance measure applied to the normalized fluorescence intensity signal from the microarray data, on a 0 to 5 scale where 0 is not present (blue) and 5 is present in high levels (red) (scale 0–5 on the bottom left of Figure). The diagram represents 125 species (HOT) in 27 samples. The patients samples grouped in two distinct clusters: Health (H1 to H12) and LAD (both moderate and severe). Separation is shown by a yellow super-imposed line. Bacterial species grouped in 5 distinct clusters (1–5, black, brown, blue, green and purple).

doi:10.1371/journal.ppat.1004698.g002

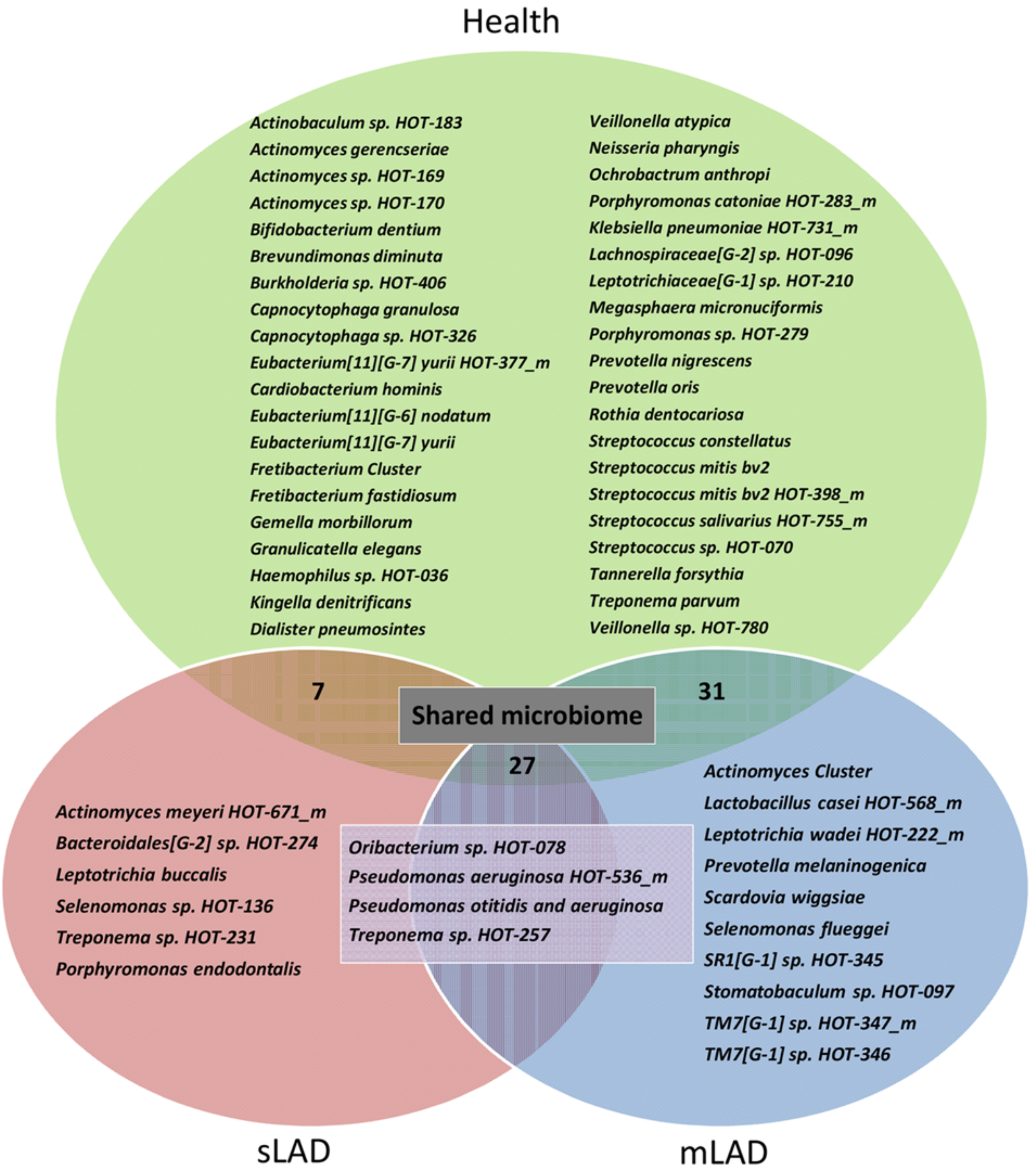

Fig 3. Unique bacterial taxa detected in health, severe and moderate LAD. Venn Diagram showing unique taxa detected in Health (n = 40, green), mLAD (n = 10, blue), sLAD (n = 6, red) and all LAD patients (n = 4, purple). Taxa (species) shared between mLAD and Health (n = 31), sLAD and Health (n = 7) or detected in all groups (n = 27) are termed a “shared microbiome” and not listed in the figure.

doi:10.1371/journal.ppat.1004698.g003

LAD-I associated communities had an enrichment in periodontitis-associated bacteria such as *Treponema* spp. found both in mLAD and sLAD, members of the TM7 phylum in mLAD and *Porphyromonas endodontalis* in sLAD. A very interesting finding in the LAD-I subgingival microbiome was the detection of *Pseudomonas aeruginosa*, a bacterium not typically found in

subgingival plaque but of potential clinical relevance in LAD where *P. aeruginosa* infections are often encountered [5]. Also notable was the detection of *Leptotrichia spp*., typically seen in health, but associated with severe infections in patients with immunodeficiency [10]. Another uniquely identified species in LAD included *Scardovia wiggsiae*, a bacterium recently associated with severe childhood caries [11] but not related to caries in our cohort where none of the patients had a single carious lesion.

To further analyze differences in microbial communities of health and moderate and severe LAD, we evaluated differences in the microbiota by level of detection. Enrichment or overgrowth of particular commensals (which are often detected at low levels in health, is the hallmark of periodontal dysbiosis in the general population [12]. In the case of sLAD, species detected higher than two fold were largely associated with periodontitis (Fig. 4A) [9]. Species with a high level of detection in sLAD included *Parvimonas micra* HOT-111, *Porphyromonas endodontalis* HOT-273_m, *Treponema maltophilum* HOT-664, *Treponema* sp. HOT-257, *Eubacterium* [11][G-3] *brachy* HOT-557 and *Bacteroidales* [G-2] sp. HOT-274. It is worth reporting that the classic "red perio-pathogenic complex" of bacteria [13], typically associated with the microbiome of periodontitis, were not detected in higher levels in LAD compared to health. Species with low detection in sLAD were health-associated commensals such as *Actinomyces naeslundii* HOT-176, *Rothia dentocariosa* HOT-587_m, *Granulicatella adiacens* HOT-534_m [9,14]. In moderate LAD the predominant feature was the reduced presence or depletion of health associated species, including the ones depleted in sLAD as well as additional health or core oral microbiome species [15,9] (Fig. 4B).

LAD-associated subgingival communities are distant from those of Aggressive Periodontitis

Our data and analysis revealed that the LAD subgingival communities are distinct from those of health, yet a meaningful comparison would be to compare the subgingival communities in LAD with those encountered in patients with aggressive periodontitis in the absence of a known immune defect such as the cohort of Localized Aggressive Periodontitis patients (LAP). To this end we obtained data from investigators that had evaluated patients who develop LAP, using the exact same HOMIM microarray approach and the same standardized microarray processing at Forsyth [16].

We evaluated the presence of highly detected bacterial species in LAP in our cohort of LAD patients (S1 Methods, S1 Fig). This comparison reveals that species highly detected in aggressive periodontitis such as *Aggregatibacter actinomycemcomitans* [17] are not detected in LAD patients. Additionally, multiple species known to be associated with both chronic and aggressive periodontitis such as *Tannerella forsythia*, *Filifactor alocis*, *Eubacterium nodatun*, *Eubacterium brachy* and *Porphyromonas gingivalis* were either undetectable in LAD or detected with lower prevalence [13,9].

Conversely, unique species detected in LAD including *Oribacterium sp*. HOT-078, *Pseudomonas aeruginosa* HOT-536_m, *Pseudomonas otitidis and aeruginosa*, and *Treponema sp.* HOT-257 were undetected in LAP [16].

Immunostimulatory potential of LAD-associated microbial communities

Having established that LAD-I periodontitis-associated microbial communities have a unique composition we wanted to gain further insight into the potential of microbial products from these communities to stimulate inflammatory responses. Periodontitis is considered a microbially-stimulated inflammatory condition. Both in chronic periodontitis and LAD-periodontitis, tooth-associated bacteria are thought to stimulate an exaggerated inflammatory response

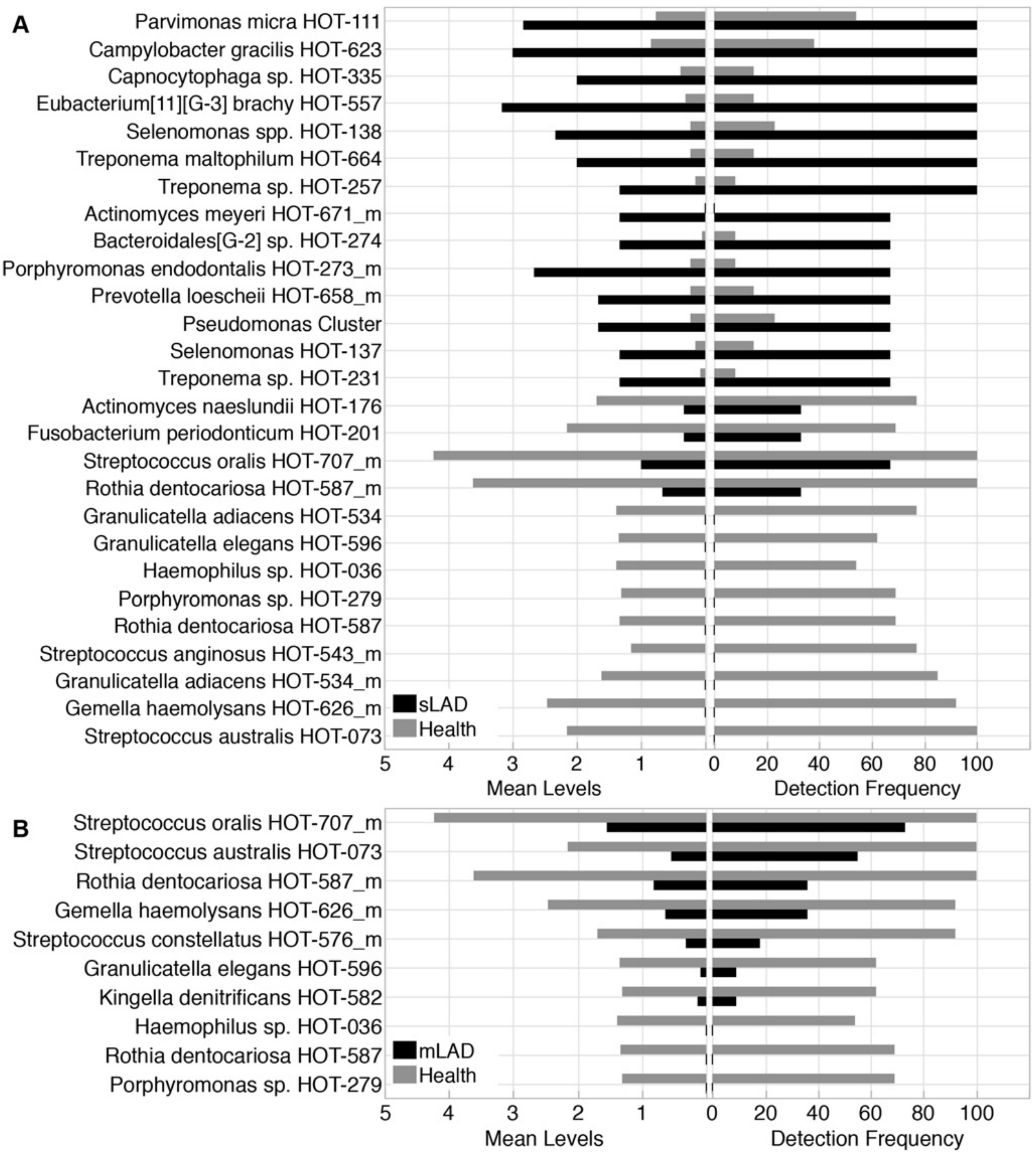

Fig 4. Bacterial taxa differentially represented in LAD and health. (A) Species with a 2 fold change or greater in severe LAD (sLAD) versus health samples. Left panel shows mean detection levels of 27 bacterial taxa that were differentially represented in severe LAD compared to health. Right panel shows detection frequency across samples of those 27 species. Percent present for each group is the number of sites that were positive for the specific species. (B) Species with a 2 fold or greater difference between mild/moderate LAD (mLAD) and health microbial communities. Left panel shows mean detection levels for 10 species that were differentially found compared to health. Right panel shows percent presence across samples for the same 10 species.

doi:10.1371/journal.ppat.1004698.g004

that leads to tissue destruction [6]. Consistent with this concept, in LAD-I we observed a significant colonization of bacteria on the tooth surface but not within the disease tissues (Fig. 5A), which are dominated by intense inflammation [6]. To investigate the participation of bacterial products stimulating the inflammatory response within the tissues of LAD periodontitis, we evaluated the presence of bacterial lipopolysaccharides (LPS) within the tissues. We found wide-spread staining for LPS within the tissue lesions of LAD-I and at a significant depth below the epithelium, but not in health. Importantly, LPS staining was present in close association with infiltrating immune cells (Fig. 5B), suggesting that bacterial cell wall components (LPS) translocating into the tissues may play a role in stimulating and exacerbating local inflammatory responses.

Human macrophages (at a concentration of $3x10^6$/ml) were left untreated (medium control) or treated with standardized amounts of inactivated subgingival plaque (equivalent of $1x10^6$ 16S rRNA gene copy number) from healthy, mLAD and LAD donors (n = 3, each) for 4 hours and processed for RNA to evaluate cytokine gene transcription. Results are shown as fold induction of mRNA expression relative to the untreated control, $p<0.05$ between health and LAD. (D) *In vivo* inoculation of murine oral mucosa with inactivated subgingival plaque from healthy volunteer (HV) and sLAD donors or purified LPS (2 μg/μl). Mice received oral injections with HV or sLAD plaque or LPS and oral tissues were harvested at 4h for evaluation IL23a at the mRNA level (n = 3). Results are shown as fold increase over untreated control, * = $p<0.05$, significant increase over treatment with HV plaque.

To evaluate the potential of microbial products from LAD-associated microbial communities to stimulate inflammatory responses, we exposed human primary macrophages to inactivated bacterial plaque from severe and moderate LAD-I donors as well as healthy volunteers. Macrophages are innate immune cells equipped to sense bacterial components and mount inflammatory responses [18,19]. Importantly, macrophages were the most abundant population of antigen presenting cells (APC) present in the inflammatory lesions of LAD-I [6]. Macrophage inflammatory cytokine responses were evaluated by measuring mRNA expression of select pro-inflammatory cytokines 4h post microbial exposure. Cytokines were selected based on their potential contribution to periodontal disease pathogenesis, including their ability to aid in the differentiation of Th1 or Th17 responses. The dominant cytokines induced in response to subgingival plaque (of health or LAD) were the two chains of the IL-23 molecule (p40 and p19). Strikingly, sLAD subgingival plaque appeared to have a significant advantage towards the induction of IL-23, a molecule previously shown to be key in the pathogenesis of LAD-I periodontitis [6] (Fig. 5C). Exposure to sLAD plaque significantly upregulated the expression of IL-23p19 *in vitro* in human macrophages compared to responses induced by healthy subgingival plaque, even when experiments were performed with a standardized microbial load for all samples. IL-23 was also induced in response to sLAD plaque in macrophages obtained from LAD patients.

To further our studies we evaluated the potential of LAD subgingival plaque to induce IL-23 mediated responses *in vivo*. For these experiments we injected standardized volumes of inactivated plaque from healthy or sLAD donors into mouse oral mucosa and harvested tissues at 4h to evaluate initial cytokine responses at the transcriptional level. Consistent with our *in vitro* observations, inoculation with sLAD subgingival plaque significantly upregulated IL-23 *in vivo*, as did purified high concentrations of LPS (Fig. 5D). Induction of LPS-triggered IL-23 responses was also relevant to our studies given the translocation of LPS into the lesional tissues.

Collectively our data show that the LAD-I associated subgingival microbial communities are distinct and bear a clear potential towards stimulating IL-23 mediated inflammatory responses.

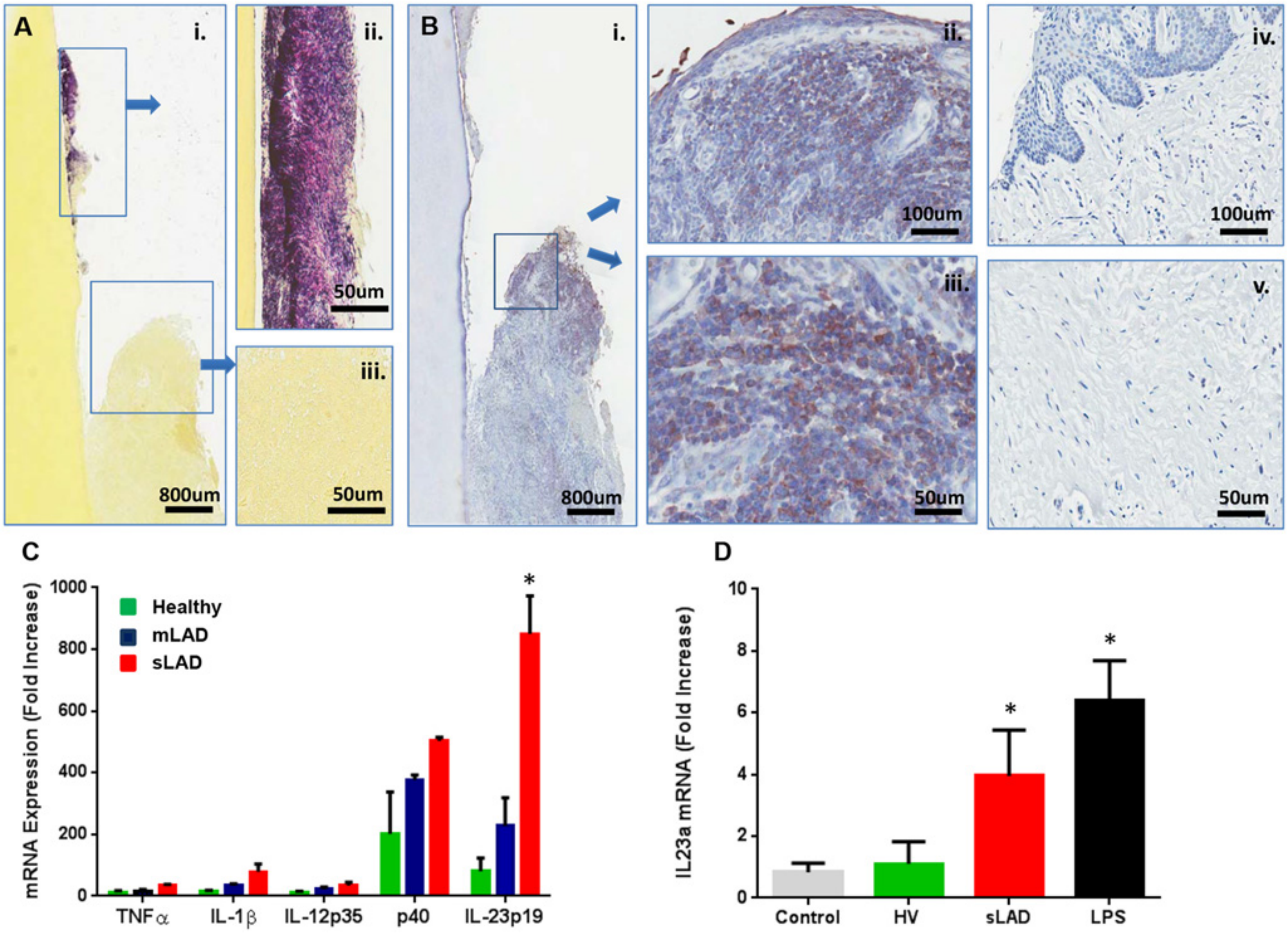

Fig 5. Immunostimulatory potential of LAD- associated dental plaque. (A) Gram staining (Brown and Brenn method) of extracted tooth and adjacent soft tissues: (i) Gram positive (violet) and negative (pink) staining on the root surface (ii) and surrounding tissues (iii)(scale bars shown). Representative of 3 LAD patients with tooth extractions. (B) Immunohistochemistry for bacterial lipopolysaccharide (LPS) on extracted LAD tooth (i) and surrounding tissues (ii, iii) as well as in healthy gingiva (iv, v). Positive staining is brown and indicated by arrows (scale bars shown). Representative of 3 LAD patients. (C) Exposure of human macrophages to LAD microbial plaque.

doi:10.1371/journal.ppat.1004698.g005

Discussion

One of the hallmarks of LAD-I is the presentation of severe periodontitis at a young age that often becomes unresponsive to treatment and leads to loss of the entire dentition [5,1]. The inflammatory component of LAD-I periodontitis was recently characterized in detail and is governed by IL-23/17 inflammatory signals driving tissue destruction [6]. Microbial signals from the tooth adherent subgingival plaque are considered to be the triggers for this dysregulated immune response. In fact, our clinical observations suggest that inflammation persists in LAD patients in the presence of a tooth adherent biofilm, but complete resolution and mucosal healing occurs following tooth extraction and removal of the local nidus of microbes.

Our current study characterized the subgingival microbiome in LAD and evaluated its potential to trigger local inflammatory responses. To date, this is the first comprehensive evaluation of the subgingival microbial communities in LAD-periodontitis. Previous studies of LAD cases had used culture based approaches and reported the presence of *Capnocytophaga*, *Eikenella corrodens* and *Candida albicans* in periodontal pockets of LAD patients [20].

Our first observation was the high biomass in the LAD subgingival biofilms. Whether this overgrowth of bacteria is related to the deficient neutrophil surveillance in LAD or is a result of the exacerbated inflammatory milieu [21] cannot be explored with our current data. However, inhibition of inflammation in mice mimicking the LAD phenotype resulted in diminished periodontal bacterial load, comparable to that of mice without neutrophil defects. This observation suggests that the inflammatory environment contributes more to the increase in bacterial load than the absence of neutrophil surveillance. Consistent with this, a previous study evaluating microbial load in chronic periodontitis had documented a significantly increased microbial load (comparable to that seen in our mLAD patients) only in sites with active bleeding and inflammation [9]. Under conditions of inflammation additional nutrients become available [22], but also the ecological environment drastically changes, thereby selective for bacteria that thrive under relevant conditions (recently defined as "*inflammophylic*" [21]) to survive and grow.

Subgingival communities in LAD-I also differ significantly from those in health in terms of microbial composition. One unifying characteristic of the LAD microbiome (both mLAD and sLAD) is the depletion of species typically associated with health. Whether this depletion represents a unique feature of LAD and its inflammatory environment or is associated with chronic use of antibiotics in these patients, or both cannot be determined. Species such as *Actinomyces naeslundii* HOT-176, *Rothia dentocariosa* HOT-587 and *Granulicatella adiacens* HOT-534, typically detected in healthy subjects by a number of studies using various approaches [9,14], were undetectable in the LAD-I microbial communities. Whether these bacteria are simply compatible with an ecological environment of health and/or participate in the maintenance of health in the oral cavity has not been explored. Health associated communities in the gastrointestinal tract have been shown to promote health and homeostasis while protecting from infection in a variety of ways [23]. Intestinal commensals directly inhibit growth of pathogenic bacteria [24], activate innate immune mechanisms to suppress competing microbes [25], tolerate commensals [26] and stimulate responses that strengthen the epithelial barrier (e.g., IL-22) [23]. It is conceivable that oral health-associated commensals may have similar properties and therefore alterations in their presence may underlie aberrant immune responses. In fact alterations in the composition of commensals (dysbiosis) is the hallmark of periodontitis in the general population [12,27].

Nevertheless, the microbial communities observed in LAD are not identical to those described in classic chronic or aggressive periodontitis. Our direct comparison of the LAD subgingival microbiome with that of patients with localized aggressive periodontitis demonstrated that species associated with aggressive periodontitis were absent or detected with low prevalence in the LAD cohort. *Aggregatibacter actinomycetemcomitans* (*Aa*), the organism most associated with aggressive periodontitis [17,28] was absent in LAD, but also multiple species associated with both chronic and aggressive periodontitis were either undetectable in LAD or detected with low prevalence [13,9]. Not consistent with a classic periodontitis-related community was the decreased number of species detected in the LAD subgingival microbiome. Although total microbial diversity cannot be assessed using a microarray approach, our comprehensive evaluation of known oral species and select non-oral microbes pointed to a decreased complexity of the LAD subgingival communities. Typically, increased microbial diversity has been documented in the majority of studies of periodontitis [13,14,9], but recently a study of metatranscriptomics in periodontitis has reported decreased richness and diversity in diseased sites [29].

How chronic antibiotic use in many of our patients may have affected the LAD microbiome is not clear. In is important to point out that despite sporadic or consistent use of antibiotics in our patients, the biomass of the LAD microbiome remained very high, suggesting a level of resistance to antibiotics in this complex biofilm [30]. It is also important to note that the LAD subingival microbiome, despite exposure to antibiotics, represents a continuous trigger for

local immunopathology. It is of course conceivable that any microbial stimulus even from a non-classical periodontitis-related community detected in LAD may be pathogenic in the setting of immune dysregulation. When immune regulation is impaired, commensals may be capable of causing disease that could not be present in another setting [23,31]. A notable example is that of mice defective in IL-10 and TGFβR2 signaling develop spontaneous colitis in response to commensals [32].

A unique species detected in LAD was *Pseudomonas aeruginosa*, a bacterium not typically harbored in subgingival plaque, that is associated with severe infections in immunocompromised hosts including LAD patients [3]. Consistent with our findings, previous reports also recovered *Pseudomonas aeruginosa* from subgingival plaque [33] and oral ulcers of LAD patients [34]. *P. aeruginosa* has unique capabilities in forming biofilms and gaining antibiotic resistance [35,36]. It can use a broad spectrum of nutrients and survive in harsh environments and is most typically encountered as a hospital acquired infection. *Pseudomonas aeruginosa* skin infections have also been reported in LAD patients [37]. How colonization with *P. aeruginosa* in the oral cavity may relate to systemic infections is unclear. Interestingly *Pseudomonas aeruginosa* infection has been linked to IL-23 dependent inflammation in a mouse model of lung infection [38], but also patients with Cystic Fibrosis [39].

Another potential pathogen for LAD patients detected in our cohort was *Leptotrichia buccalis* as well as multiple other *Leptotrichia spp*. While these are considered commensals, *L. buccalis* has been linked to bacteremia and severe illness in neutropenic and immunocompromised patients [10] [40].

How LAD-associated microbial communities can be linked to the local inflammatory responses is difficult to determine. However, our observation of LPS translocation within the inflammatory lesions in LAD-I, suggests that bacterial products participate in the local immunopathology. Bacterial LPS within tissues has been previously associated with local and systemic immune activation, particularly in the setting of immunodeficiency such as chronic HIV infection [41,42,43].

To further explore the potential of LAD-associated microbial communities stimulating inflammation, we studied their potential in triggering cytokine responses *in vitro and in vivo*. For this purpose we performed stimulations *in vitro* in human primary macrophages, the dominant APC population in LAD-I and in a murine setting *in vivo* with microbial components from LAD plaque. We found that innate responses to human subgingival plaque and particularly LAD-associated plaque are dominated by an IL-23 cytokine signature. These data complement our previous immunological findings in the tissues of LAD-associated periodontitis where IL-23/IL-17 are the dominant cytokines observed. Our data suggest that microbial stimuli from the dental plaque can trigger an IL-23/IL-17 response which appears to be exaggerated in LAD patients due to the absence of neutrophils in the tissues regulating the IL-17 axis [43,6]. Taken together with our previous report [6], the current findings support the hypothesis that the IL-23/IL-17 axis is a critical target in LAD-periodontitis.

Materials and Methods

Diagnostics and clinical data

Patients were diagnosed with LAD-I based on defined biallelic CD18 mutations and flow cytometric analysis of CD18 expression on peripheral neutrophils, as previously described [4]. Patients were designated as severe LAD (sLAD) or moderate LAD (mLAD) on the basis of percentage of CD18 expression and clinical manifestations. Healthy subjects had no significant medical history, tested negative for HIV (Human Immunodeficiency Virus), HBV (Hepatitis B Virus), HCV (Hepatitis C Virus) and had HbA1c<6% (Hemoglobin 1c).

Ethics statement

Clinical data and demographics for all patients were obtained from NIH records. All research and procedures involving human subjects (including use of patient medical data and collection of patient oral samples and blood) were reviewed and approved by the Institutional Review Boards of the National Institute of Allergy and Infectious Diseases and the National Institute of Dental and Craniofacial Research. All patients or guardians provided written informed consent for participation. All adult participants provided their own consent. Data and samples were anonymized (de-identified) for relevant analysis.

Oral/periodontal examination and microbial sampling of subgingival plaque

Periodontal evaluation included measurements of loss of tooth-supporting structures (connective tissue and bone, measured as Clinical Attachment Loss = CAL and Probing depth = PD) in millimeters. Measurements were performed using a periodontal probe calibrated in mm, at six sites per tooth in all teeth on each patient and expressed as mean measurements per patient [44]. Clinical parameters in sites sampled for subgingival microbiome were recorded separately. Tooth adherent bacterial plaque was sampled from areas below the gum line (subgingival) with a standardized approach to avoid sampling variability [9]. Briefly, samples were collected from all subjects by a single clinician (NM) to avoid intra-examiner variability. Sampling was performed from the same index teeth in all patients at the identical intraproximal areas, using a mini five gracey curette and a single stroke technique. Dental plaque samples from each site were placed in TE buffer, homogenized by vortexing, and divided in two parts. One part used for DNA isolation and the other part for *in vitro* functional assays.

DNA isolation, bacterial quantification and HOMIM

Bacterial DNA was extracted from human tooth-associated subgingival biofilm (dental plaque) samples using the MasterPure DNA Extraction Kit (Epicentre Biotechnologies, Madison, WI).

Real-time PCR assays were performed to determine total bacterial load using 16S rRNA universal primers, as previously described [9]. Briefly, standard curves were prepared using 10-fold dilutions of genomic DNA of *Streptococcus gordonii* ATCC 35105 ranging from 10^2 to 10^8 16S rRNA molecules. PCR reactions were performed in a final volume of 20 μl per reaction, containing 10 μl of TaqMan Gene Expression Master Mix (Applied Biosystems/Life Technologies, Grand Island, NY), 900 nM of each primer pair, 250 nM of the fluorogenic probe, 1 μl DNA template and PCR water. Amplification was carried out in a 7500 Real-Time PCR system (Applied Biosystems/Life Technologies) and the thermocycler conditions were 50°C for 2 min, 95°C 10 min and 40 cycles of 95°C for 15 s and 60°C for 1 min. Bacterial load from multiple sites of the each patient were first averaged to obtain one value per patient. Average patient values were used for group comparisons (health vs mLAD and health vs sLAD).

For Microarray analysis, total genomic DNA was submitted to The Forsyth Institute for HOMIM (Human Oral Microbe Identification Microarray) [14]. Briefly, 16S rRNA genes were PCR-amplified from DNA extracts with 16S rRNA gene universal forward and reverse primers and labeled via incorporation of Cy3-dCTP in a second nested PCR. HOMIM uses 16S rRNA-based, reverse-capture oligonucleotide probes (typically 18 to 20 bases), for approximately 300 taxa. Detection of a particular taxon is determined by the presence of a fluorescent spot for that unique probe. A mean intensity for each taxon was calculated from hybridization spots of the same probe and signals were normalized using universal probes and calculated as described previously [14]. Any original signal that was less than two times the background value was re-

set to 0. Signals greater than 1 were categorized into scores from 1 to 5 corresponding to ranked signal levels [14].

HOMIM statistical analysis

Data annotation. The data matrix consisted of 379 probes and 27 samples; from healthy (n = 13 sites from twelve patients) and LAD-I (n = 14 sites from five patients) donors. LAD-I samples were divided into a severe, sLAD (n = 3 sites from one patient) and a mild/moderate, mLAD group (n = 11 sites from four patients). The data were filtered to remove any probes that had an intensity value of 0 across all samples. Probe annotations containing multiple Human Oral Taxon (HOT) ID's were designated with a "_m" appended to the HOT ID name. The complete list for these "_m" HOT ID is presented in S2 Table. The intensity values for multiple probes having the same HOT ID were averaged. The final data matrix used for analysis consisted of 158 unique HOT ID's. Throughout the manuscript, the terms HOT and species will be used interchangeably for simplicity.

Principal component analysis. Principal component analysis was performed on the intensity value calls of 158 unique HOT and 27 subgingival samples. Principal component 1 (PC1) was plotted versus principal component 2 (PC2). The first two PC's represent 35% of the total variability in the data matrix. Ellipses are drawn around at least 50 percent of the samples from each of the 3 sample groups.

Data filtering and transformation. The data were submitted to hierarchical clustering using Ward method (JMP statistical discovery software, Cary, NC). Included HOT had to be designated present in at least two samples within any of the three groups. A HOT is deemed present if its intensity value is 1 or greater. This filter removed 33 HOT from the data matrix, generating a matrix of 125 HOT for further analysis. A Venn diagram was created for those 125 HOT passing this filter in order to study the relationship between patient groups for HOT that were present. In order to determine total number of HOT detected per site, intensity values were changed to a binary variable (1 or 0, with 0 representing no detection and 1 assigned to detected HOT) and the sum was calculated across all 125 binary HOT. The detection frequency is defined by calculating the mean of the binary data per group then multiplying by 100.

To select HOT of interest with a twofold or greater difference in level of detection between two groups, the logarithm base 10 transform was applied to the group means and the difference between the mean of the log values was calculated. Because this data is so sparse, a value of 1 was added to all group means before the logarithm was taken in order to alleviate the problem of taking the log of 0. Comparisons were performed on the averaged individual patient data.

Statistical testing

A one-way analysis of variance (ANOVA) comparing three groups (health, mLAD and sLAD) was conducted on individual patient data from bacterial loads and total HOT detected from the microarray. Subsequently 3 post-hoc t-tests comparing any two groups were applied for individual comparisons. Separate sampling data points taken from the same patient were averaged and that average was used to represent an individual patient. Total loads data was found to be significant at $p<.05$ while the total HOT detected on the microarray data was not significant ($p = 0.11$).

Histology and immunohistochemistry

Biopsy specimens (tooth and surrounding soft tissues) were formalin-fixed, decalcified and then embedded in paraffin. Sections of 5-μm were obtained and stained with a modified Brown and Brenn (B&B) protocol [45] to evaluate the presence of Gram-positive/negative bacteria in

tissues. Immunohistochemical staining for LPS was performed as previously described [46]. Briefly, tissue slides were deparaffinized and hydrated with graded alcohols. Antigen retrieval was carried out by heating sections in 1X DIVA Declocker reagent (Biocare Medical, Concord, CA) and then cooled down to room temperature. Slides were incubated in 3% H_2O_2 for 25 min, to block the endogenous peroxidase, washed and then incubated with Background Sniper blocking reagent (Biocare Medical, Concord, CA) followed by another blocking step with bovine serum albumin. Incubation with primary antibody to LPS-core (Hycult biotech, Plymouth Meeting, PA) was performed for 1 hour at room temperature, washed with PBS, immunolabeled with the ImmPRESS detection system (Vector, Burlingame, CA) and developed with ImmPACT DAB peroxidase substrate (Vector, Burlingame, CA). Finally, tissue slides were counterstained with Mayer's hematoxylin, dehydrated and mounted with Permount (Fisher Scientific, Pittsburg, PA). All sections were scanned using the Aperio ScanScope system (Leica Biosystems, Buffalo Grove, IL).

Macrophage cell culture and stimulation with dental plaque

Human peripheral blood mononuclear cells (PBMC) obtained from healthy volunteers at the Department of Transfusion Medicine (National Institutes of Health, Bethesda, MD) were diluted in endotoxin-free PBS without Ca^{2+} and Mg^{2+} (Life Technologies, Grand Island, NY) and density-sedimented on lymphocyte separation medium (LSM; ICN Pharmaceuticals, Aurora, OH). Monocytes were purified from the mononuclear cell layer using centrifugal elutriation and plated for the generation of macrophages as previously described [47,18].

Macrophages (at a concentration of $3x10^6$/ml) were left untreated (medium control) or treated with *Escherichia coli* LPS (1, 10 and 100ng/ml Sigma, St. Louis, MO) or subgingival plaque from healthy and LAD donors. For these assays total subgingival plaque was removed from a given site, placed in 100ul of TE buffer, homogenized by vortexing and heat/freeze inactivated. Macrophage cultures were exposed to a standardized amount of total bacterial DNA ($1x10^6$16S rRNA gene copy number) of plaque for 4 h after which macrophage cultures were processed for RNA to evaluate cytokine gene transcription.

In vivo inoculation with subgingival plaque

C57BL/6J mice were anesthetized with ketamine/xylazine (80 mg/kg and 5 mg/kg) and thereafter received oral mucosal injections with LPS (2 μg/μl, *E. coli* LPS, serotype 055:B5, Sigma, St. Louis, MO) or inactivated subgingival plaque from healthy volunteers or from sLAD patients. Oral tissues were harvested at 4h for tissue processing. Tissues were placed in TRIzol Reagent (Life Technologies, Carlsbad, CA) and homogenized using the miltenyi gentleMACS dissociator along with gentleMACS M tubes from miltenyibiotec (San Diego, CA).

RNA isolation and real-time PCR

Total RNA was isolated from myeloid cells with the RNeasy Mini Columns (Qiagen, Valencia, CA). RNA was reverse transcribed using an oligodeoxythymidylic acid primer and the resulting cDNA amplified by real-time PCR on an ABI Prism 7500 Sequence Detector (Applied Biosystems/Life Technologies). Amplification was performed with human TaqMan inventoried expression assays for TNFα (hs00174128_m1), IL12A (p35) (hs00168405_m1), IL12B (p40) (hs00233688_m1), IL23a (hs00900828_g1), IL1β (hs01555410_m1) and IL6 (hs00174131_m1) and mouse IL23a (Mm01160011_g1) and HPRT (Mm01545399_m1), (Applied Biosystems/ Life Technologies). Amplification parameters and conditions were set by the manufacturer. Data were analyzed using the $2^{-\Delta\Delta CT}$ method ($2^{\wedge((-1)*(\text{Mean cycle number of Target-mean cycle number of housekeeping gene}))}$) and expressed as fold induction relative to control.

Supporting Information

S1 Methods. Comparison of prevalence for select species in LAD and Localized Aggressive Periodontitis.
(DOCX)

S1 Fig. Detection of species highly prevalent in Localized Aggressive Periodontitis. Frequency of detection of HOTs in the cohorts of localized aggressive periodontitis patients (LAP, n = 7, black) and LAD-I (n = 5, grey). HOTs shown are those highly detected in LAP patients only [16]. Frequency of Detection corresponds to % present in each cohort.
(TIF)

S1 Table. Clinical characteristics of the LAD cohort.
(DOCX)

S2 Table. HOT corresponding to multiple species.
(DOCX)

S3 Table. Cluster identification and detection frequency for each.
(DOCX)

Acknowledgments

The authors wish to thank Dr Patricia Diaz and Dr George Hajishengallis for critically reviewing the manuscript.

Author Contributions

Conceived and designed the experiments: NMM SMH. Performed the experiments: LA TGW BJP ND NMM. Analyzed the data: NIC JJB PJM DHF NMM. Contributed reagents/materials/analysis tools: BJP DHF GU. Wrote the paper: NMM SMH.

References

1. Rosenzweig SD, Holland SM (2004) Phagocyte immunodeficiencies and their infections. J Allergy Clin Immunol 113: 620–626. PMID: 15100664
2. Hanna S, Etzioni A (2011) New host defense mechanisms against Candida species clarify the basis of clinical phenotypes. J Allergy Clin Immunol 127: 1433–1437. doi: 10.1016/j.jaci.2011.03.026 PMID: 21497889
3. Gerald L. Mandell JEB, and Raphael Dolin (2009) Mandell, Douglas, and Bennett's Principles and Practice of Infectious Diseases In: Gerald L. Mandell JEB, and Raphael Dolin, editor. Mandell, Douglas, and Bennett's Principles and Practice of Infectious Diseases Philadelphia: Elsevier. doi: 10.1056/NEJMoa1304839.Two PMID: 24450891
4. Uzel G, Tng E, Rosenzweig SD, Hsu AP, Shaw JM, et al. (2008) Reversion mutations in patients with leukocyte adhesion deficiency type-1 (LAD-1). Blood 111: 209–218. PMID: 17875809
5. Hanna S, Etzioni A (2012) Leukocyte adhesion deficiencies. Ann N Y Acad Sci 1250: 50–55. doi: 10.1111/j.1749-6632.2011.06389.x PMID: 22276660
6. Moutsopoulos N (2014) Defective neutrophil recruitment in leukocyte adhesion deficiency causes local IL-17–driven inflammatory bone loss. Science Translational Medicine 6: 40.
7. Hajishengallis E, Hajishengallis G (2014) Neutrophil homeostasis and periodontal health in children and adults. J Dent Res 93: 231–237. doi: 10.1177/0022034513507956 PMID: 24097856
8. Armitage GC (1999) Development of a classification system for periodontal diseases and conditions. Ann Periodontol 4: 1–6. PMID: 10863370
9. Abusleme L, Dupuy AK, Dutzan N, Silva N, Burleson JA, et al. (2013) The subgingival microbiome in health and periodontitis and its relationship with community biomass and inflammation. ISME J 7: 1016–1025. doi: 10.1038/ismej.2012.174 PMID: 23303375

10. Reig M, Baquero F, Garcia-Campello M, Loza E (1985) Leptotrichia buccalis bacteremia in neutropenic children. J Clin Microbiol 22: 320–321. PMID: 4031045

11. Tanner AC, Mathney JM, Kent RL, Chalmers NI, Hughes CV, et al. (2011) Cultivable anaerobic microbiota of severe early childhood caries. J Clin Microbiol 49: 1464–1474. doi: 10.1128/JCM.02427-10 PMID: 21289150

12. Hajishengallis G (2014) Immunomicrobial pathogenesis of periodontitis: keystones, pathobionts, and host response. Trends Immunol 35: 3–11. doi: 10.1016/j.it.2013.09.001 PMID: 24269668

13. Socransky SS, Haffajee AD, Cugini MA, Smith C, Kent RL Jr. (1998) Microbial complexes in subgingival plaque. J Clin Periodontol 25: 134–144. PMID: 9495612

14. Colombo AP, Boches SK, Cotton SL, Goodson JM, Kent R, et al. (2009) Comparisons of subgingival microbial profiles of refractory periodontitis, severe periodontitis, and periodontal health using the human oral microbe identification microarray. J Periodontol 80: 1421–1432. doi: 10.1902/jop.2009.090185 PMID: 19722792

15. Colombo AP, Bennet S, Cotton SL, Goodson JM, Kent R, et al. (2012) Impact of periodontal therapy on the subgingival microbiota of severe periodontitis: comparison between good responders and individuals with refractory periodontitis using the human oral microbe identification microarray. J Periodontol 83: 1279–1287. PMID: 22324467

16. Fine DH, Markowitz K, Fairlie K, Tischio-Bereski D, Ferrendiz J, et al. (2013) A consortium of Aggregatibacter actinomycetemcomitans, Streptococcus parasanguinis, and Filifactor alocis is present in sites prior to bone loss in a longitudinal study of localized aggressive periodontitis. J Clin Microbiol 51: 2850–2861. doi: 10.1128/JCM.00729-13 PMID: 23784124

17. Fine DH, Markowitz K, Furgang D, Fairlie K, Ferrandiz J, et al. (2007) Aggregatibacter actinomycetemcomitans and its relationship to initiation of localized aggressive periodontitis: longitudinal cohort study of initially healthy adolescents. J Clin Microbiol 45: 3859–3869. PMID: 17942658

18. Nares S, Moutsopoulos NM, Angelov N, Rangel ZG, Munson PJ, et al. (2009) Rapid myeloid cell transcriptional and proteomic responses to periodontopathogenic Porphyromonas gingivalis. Am J Pathol 174: 1400–1414. doi: 10.2353/ajpath.2009.080677 PMID: 19264901

19. Hasturk H, Kantarci A, Van Dyke TE (2012) Oral inflammatory diseases and systemic inflammation: role of the macrophage. Front Immunol 3: 118. doi: 10.3389/fimmu.2012.00118 PMID: 22623923

20. Cox DP, Weathers DR (2008) Leukocyte adhesion deficiency type 1: an important consideration in the clinical differential diagnosis of prepubertal periodontitis. A case report and review of the literature. Oral Surg Oral Med Oral Pathol Oral Radiol Endod 105: 86–90. PMID: 17618138

21. Hajishengallis G (2014) The inflammophilic character of the periodontitis-associated microbiota. Mol Oral Microbiol.

22. Socransky SS, Haffajee AD (2005) Periodontal microbial ecology. Periodontol 2000 38: 135–187.

23. Littman DR, Pamer EG (2011) Role of the commensal microbiota in normal and pathogenic host immune responses. Cell Host Microbe 10: 311–323. doi: 10.1016/j.chom.2011.10.004 PMID: 22018232

24. Fukuda S, Toh H, Hase K, Oshima K, Nakanishi Y, et al. (2011) Bifidobacteria can protect from enteropathogenic infection through production of acetate. Nature 469: 543–547. doi: 10.1038/nature09646 PMID: 21270894

25. Ivanov II, Atarashi K, Manel N, Brodie EL, Shima T, et al. (2009) Induction of intestinal Th17 cells by segmented filamentous bacteria. Cell 139: 485–498. doi: 10.1016/j.cell.2009.09.033 PMID: 19836068

26. Atarashi K, Tanoue T, Shima T, Imaoka A, Kuwahara T, et al. (2011) Induction of colonic regulatory T cells by indigenous Clostridium species. Science 331: 337–341. doi: 10.1126/science.1198469 PMID: 21205640

27. Darveau RP (2010) Periodontitis: a polymicrobial disruption of host homeostasis. Nat Rev Microbiol 8: 481–490. doi: 10.1038/nrmicro2337 PMID: 20514045

28. Shaddox LM, Huang H, Lin T, Hou W, Harrison PL, et al. (2012) Microbiological characterization in children with aggressive periodontitis. J Dent Res 91: 927–933. PMID: 22863892

29. Jorth P, Turner KH, Gumus P, Nizam N, Buduneli N, et al. (2014) Metatranscriptomics of the human oral microbiome during health and disease. MBio 5: e01012–01014. doi: 10.1128/mBio.01012-14 PMID: 24692635

30. Davies D (2003) Understanding biofilm resistance to antibacterial agents. Nat Rev Drug Discov 2: 114–122. PMID: 12563302

31. Elinav E, Strowig T, Kau AL, Henao-Mejia J, Thaiss CA, et al. (2011) NLRP6 inflammasome regulates colonic microbial ecology and risk for colitis. Cell 145: 745–757. doi: 10.1016/j.cell.2011.04.022 PMID: 21565393

32. Bloom SM, Bijanki VN, Nava GM, Sun L, Malvin NP, et al. (2011) Commensal Bacteroides species induce colitis in host-genotype-specific fashion in a mouse model of inflammatory bowel disease. Cell Host Microbe 9: 390–403. doi: 10.1016/j.chom.2011.04.009 PMID: 21575910

33. Roberts MW, Atkinson JC (1990) Oral manifestations associated with leukocyte adhesion deficiency: a five-year case study. Pediatr Dent 12: 107–111. PMID: 2133935

34. Majorana A, Notarangelo LD, Savoldi E, Gastaldi G, Lozada-Nur F (1999) Leukocyte adhesion deficiency in a child with severe oral involvement. Oral Surg Oral Med Oral Pathol Oral Radiol Endod 87: 691–694. PMID: 10397659

35. Lyczak JB, Cannon CL, Pier GB (2000) Establishment of Pseudomonas aeruginosa infection: lessons from a versatile opportunist. Microbes Infect 2: 1051–1060. PMID: 10967285

36. Harmsen M, Yang L, Pamp SJ, Tolker-Nielsen T (2010) An update on Pseudomonas aeruginosa biofilm formation, tolerance, and dispersal. FEMS Immunol Med Microbiol 59: 253–268. doi: 10.1111/j.1574-695X.2010.00690.x PMID: 20497222

37. Mellouli F, Ksouri H, Barbouche R, Maamer M, Hamed LB, et al. (2010) Successful treatment of Fusarium solani ecthyma gangrenosum in a patient affected by leukocyte adhesion deficiency type 1 with granulocytes transfusions. BMC Dermatol 10: 10. doi: 10.1186/1471-5945-10-10 PMID: 20929531

38. Dubin PJ, Kolls JK (2007) IL-23 mediates inflammatory responses to mucoid Pseudomonas aeruginosa lung infection in mice. Am J Physiol Lung Cell Mol Physiol 292: L519–528. PMID: 17071720

39. McAllister F, Henry A, Kreindler JL, Dubin PJ, Ulrich L, et al. (2005) Role of IL-17A, IL-17F, and the IL-17 receptor in regulating growth-related oncogene-alpha and granulocyte colony-stimulating factor in bronchial epithelium: implications for airway inflammation in cystic fibrosis. J Immunol 175: 404–412. PMID: 15972674

40. Couturier MR, Slechta ES, Goulston C, Fisher MA, Hanson KE (2012) Leptotrichia bacteremia in patients receiving high-dose chemotherapy. J Clin Microbiol 50: 1228–1232. doi: 10.1128/JCM.05926-11 PMID: 22205794

41. Brenchley JM, Price DA, Schacker TW, Asher TE, Silvestri G, et al. (2006) Microbial translocation is a cause of systemic immune activation in chronic HIV infection. Nat Med 12: 1365–1371. PMID: 17115046

42. Kristoff J, Haret-Richter G, Ma D, Ribeiro RM, Xu C, et al. (2014) Early microbial translocation blockade reduces SIV-mediated inflammation and viral replication. J Clin Invest 124: 2802–2806. doi: 10.1172/JCI75090 PMID: 24837437

43. Stark MA, Huo Y, Burcin TL, Morris MA, Olson TS, et al. (2005) Phagocytosis of apoptotic neutrophils regulates granulopoiesis via IL-23 and IL-17. Immunity 22: 285–294. PMID: 15780986

44. (2000) Parameter on comprehensive periodontal examination. American Academy of Periodontology. J Periodontol 71: 847–848. PMID: 10875687

45. Taylor RD (1966) Modification of the Brown and Brenn gram stain for the differential staining of gram-positive and gram-negative bacteria in tissue sections. Am J Clin Pathol 46: 472–474. PMID: 4162586

46. Estes JD, Harris LD, Klatt NR, Tabb B, Pittaluga S, et al. (2010) Damaged intestinal epithelial integrity linked to microbial translocation in pathogenic simian immunodeficiency virus infections. PLoS Pathog 6: e1001052. doi: 10.1371/journal.ppat.1001052 PMID: 20808901

47. Moutsopoulos NM, Kling HM, Angelov N, Jin W, Palmer RJ, et al. (2012) Porphyromonas gingivalis promotes Th17 inducing pathways in chronic periodontitis. J Autoimmun 39: 294–303. doi: 10.1016/j.jaut.2012.03.003 PMID: 22560973
